# Refining the study of decision-making in animals: differential effects of d-amphetamine and haloperidol in a novel touchscreen-automated Rearing-Effort Discounting (RED) task and the Fixed-Ratio Effort Discounting (FRED) task

**DOI:** 10.1038/s41386-023-01707-z

**Published:** 2023-08-29

**Authors:** Laura Lopez-Cruz, Benjamin U. Phillips, Jonathan M. Hailwood, Lisa M. Saksida, Christopher J. Heath, Timothy J. Bussey

**Affiliations:** 1 Department of Psychology and MRC/Wellcome Trust Behavioural and Clinical Neuroscience Institute, University of Cambridge, Downing Street, Cambridge, CB2 3EB UK; 2 Robarts Research Institute and Department of Physiology and Pharmacology, Schulich School of Medicine and Dentistry, Western University, London, ON N6A 5C1 Canada; 3 School of Life, Health and Chemical Sciences, The Open University, Walton Hall, Milton Keynes, MK7 6AA UK; 4 Present Address: School of Life, Health and Chemical Sciences, The Open University, Walton Hall, Milton Keynes, MK7 6AA UK

**Keywords:** Motivation, Reward, Pharmacology

## Abstract

Effort-based decision-making is impaired in multiple psychopathologies leading to significant impacts on the daily life of patients. Preclinical studies of this important transdiagnostic symptom in rodents are hampered, however, by limitations present in currently available decision-making tests, including the presence of delayed reinforcement and off-target cognitive demands. Such possible confounding factors can complicate the interpretation of results in terms of decision-making per se. In this study we addressed this problem using a novel touchscreen Rearing-Effort Discounting (RED) task in which mice choose between two single-touch responses: rearing up to touch an increasingly higher positioned stimulus to obtain a High Reward (HR) or touching a lower stimulus to obtain a Low Reward (LR). To explore the putative advantages of this new approach, RED was compared with a touchscreen version of the well-studied Fixed Ratio-based Effort Discounting (FRED) task, in which multiple touches are required to obtain an HR, and a single response is required to obtain an LR. Results from dopaminergic (haloperidol and d-amphetamine), behavioral (changes in the order of effort demand; fixed-ratio schedule in FRED or response height in RED), and dietary manipulations (reward devaluation by pre-feeding) were consistent with the presence of variables that may complicate interpretation of conventional decision-making tasks, and demonstrate how RED appears to minimize such variables.

## Introduction

Effort-based decision making is impaired in multiple psychopathologies, including depression, schizophrenia and Parkinson’s disease [1–4]. Animal models of effort-based decision-making are essential for understanding and developing treatments for these disorders. In the last few decades, several tasks for assessing this process have been developed in animals [5–12], some of them forward-translated to humans [1–3, 13]. Effort-based decision-making tasks present situations in which animals choose between two options that require different levels of effort expenditure and result in the delivery of two qualitatively and/or quantitatively different reinforcers [5, 14–17]. However, in many decision-making tasks interpretation of performance in terms of cost/benefit decision-making per se can be hampered by the presence of off-target cognitive demands. Floresco et al. [16, 18], for example, using a two-lever choice fixed ratio (FR)-based Effort Discounting task, observed that engaging in a High Effort (HE) FR response incurs an unintended delay between choice and the preferred reward (e.g., between the first lever press and the last one reinforced) which is not present after the Low Effort (LE) but less preferred option, which requires only one lever press. Such possible confounds make it difficult to ascertain whether a given manipulation has effects on effort-based or delay-based evaluations [18], processes that engage very different brain regions [19, 20]. Moreover, it has been reported that the order in which different effort conditions are presented within a session—ascending or descending—can make a substantial difference to the cognitive demands of the task and what neural systems are recruited. Indeed, some decision-making tasks using fixed-ratio designs featuring descending rather than ascending effort demand fail to show even the basic expected discounting profile [18], and interventions such as drugs can show very different effects depending on the order of demand presentation [18, 21, 22]. These findings indicate that in addition to decision-making per se, these tasks present off-target cognitive demands such as the requirement of the subject to calculate, infer, or remember the effort associated with two identical stimuli in the presence of changing levels of effort [18]. This problem also impacts on the translatability of research using these tasks: human studies often use designs in which effort conditions are presented in a random manner and with effort cues that make explicit the effort required on a given trial [2, 23].

It is widely reported that the dopamine (DA) system plays a critical role in regulating effort-related decision making, and systemically administered agents targeting this system have been a consistent focus of efforts aimed at discovering new treatments [18, 24–32]. However, systemic manipulations of dopamine can affect many other processes such as timing ability, tolerance to delays of reinforcement, memory, behavioral arousal or resistance to extinction [6, 19, 21, 33–35]. Thus, several studies have attempted to unravel the effects of dopaminergic manipulations on physical effort- and delay-based decision-making and have attempted to isolate drugs’ effects on motivation from their arousal components by adjusting the delays of reinforcement [18–20], comparing different efforts (e.g., repeated lever presses vs. different lever weights) [36] or combining different tasks (e.g., PR vs. Hold-down task) [6].

In the present study we developed and validated the touchscreen-based Rearing-Effort Discounting (RED) task, designed to minimize the limitations of currently available decision-making tests, thus allowing a clearer interpretation of results in terms of physical effort-based decision making. In RED, mice can choose between rearing up to touch a stimulus associated with a big reward (High Effort/High Reward (HE/HR) option) or responding to an easily reachable stimulus associated with a small reward (Low Effort/Low Reward (LE/LR) option). The HE/HR stimulus can be raised to a higher location during the test session, while the LE/LR stimulus remains fixed in a low and easily reachable location. This design allows the use of an FR1 schedule (a single response) for both options thus eliminating the potential temporal confounds associated with higher FR-based HE option requirements. Moreover, in this task animals can allocate their behavior based on explicitly visible costs and do not need to calculate, infer, or remember the effort associated with two identical stimuli associated with different and changing levels of effort. To validate RED, we tested dopaminergic drugs known to affect performance in conventional effort and delay-based decision-making tasks (haloperidol and d-amphetamine) [8, 10, 18, 37–40], their co-administration to determine RED’s suitability for assessing pharmacological rescue of a deficit model, and its sensitivity to reward devaluation by pre-feeding. Effects of the order of presentation of effort demand were also assessed (Experiment 1–3). RED was compared with a touchscreen version of the two-lever FR-based effort discounting task (Fixed-Ratio Effort Discounting (FRED)), with and without equivalent delays. Test setting, stimuli, reward and other task features were identical in RED and FRED, so the two tasks could be directly compared (Experiment 4).

## Materials and methods

### Subjects

Adult male C57BL/6 mice (*n* = 56) were used throughout (Charles River Laboratories, Margate, UK). The University of Cambridge Animal Welfare and Ethical Review Body (AWERB) reviewed and approved this research in accordance with the Animals (Scientific Procedures) Act 1986 Amendment Regulations 2012.

Details of housing conditions are provided in Supplementary Materials.

### Drugs

**Fig. 1:**
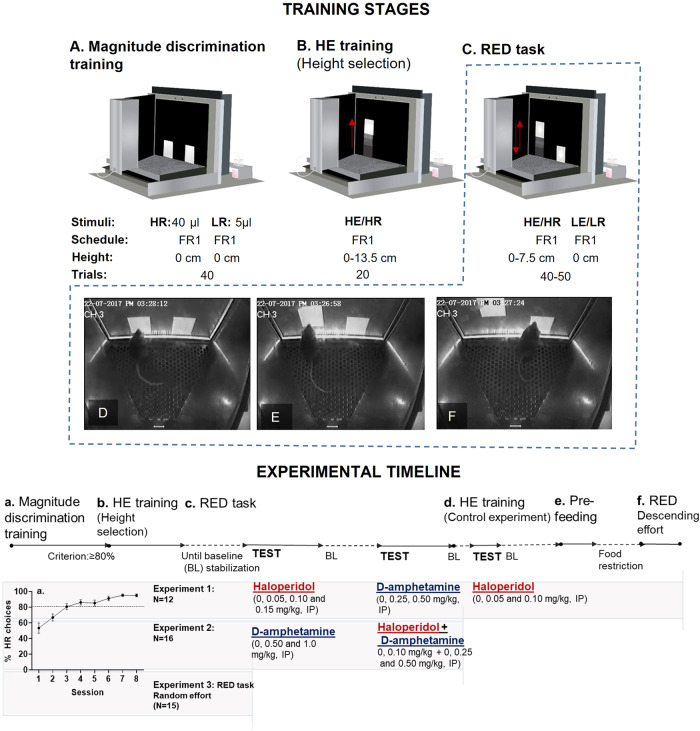
RED task training stages (top panel) and experimental timeline (bottom panel). Top panel: Mice were trained to discriminate between two reward magnitudes: a High Reward (HR) and a Low Reward (LR) (60 min session/day or until trial completion). A white rectangle was allocated for either the HR or LR delivery on the left or on the right of the screen (counterbalanced between animals). After a single-touch to the stimulus (FR1), reward delivery was accompanied by magazine illumination and a brief tone (500 ms, 3 kHz). Reward collection was followed by a 0.5 s inter-trial interval (ITI). These parameters remained constant in the remainder of the experimental stages. This training ended when a mouse chose HR on ≥80% of trials on at least 3 consecutive days (**A**). Animals were then given “High Effort (HE) training” to train them to reach up to touch the increasingly high stimulus and to select or optimize Response Height (**B**). For HE training, only the HE stimulus was presented and the required effort was gradually incremented by increasing Response Height. Response Height was increased 1.5 cm every 2 trials and 10 different Response Heights were presented (total trials = 20). The Response Heights ranged from 0 to 13.5 cm. During the three last sessions 40 s without a screen response was used as the breakpoint and the highest Response Height animals could reach was recorded and the optimal heights selected (**C**) (Supplementary Materials). In the RED task, mice could choose between the High Effort/High Reward (HE/HR) and Low Effort/Low Reward (LE/LR) stimuli (Response Height 0 cm) for 10 trials. After these 10 trials, the HE/HR stimulus was moved up while the LE/LR stimulus remained fixed in one location (Response Height 0 cm). The same sequence was repeated for different Response Heights in a descending manner (Experiment 1) or in a random order (Experiment 3) (See experimental timeline, bottom panel). Mouse choosing the HE/HR option when Response Height is 0 cm for both HE/HR and LE/LR options (**D**), mouse choosing the HE/HR stimulus when Response Height is 3 cm (**E**), and mouse choosing the LE/LR option (Response Height 0 cm) when HE/HR Response Height is 4.5 cm (**F**). Note that in (**B**) and (**C**), greyscale is used to illustrate the initial or previous HE/HR stimulus position; in practice all targets were white and only one HE stimulus was displayed in a given trial. Bottom panel: Experimental timeline (**a**–**f**).

**Fig. 2:**
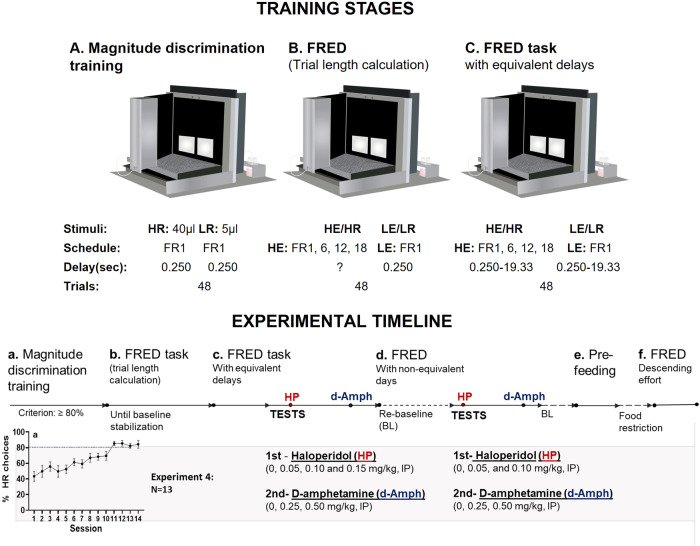
FRED task training stages (top panel) and experimental timeline (bottom panel). In magnitude discrimination training, mice received one daily session (60 min or all trials completed) until they chose the High Reward (HR) on ≥80% of trials for at least 3 consecutive sessions. Each session consisted of 48 discrete choice trials, separated into four blocks. Each block consisted of two forced-choice trials on which only one of the two stimuli (one assigned to the HR and one assigned to the Low Reward (LR)) was presented in randomized order, and 10 free-access choice trials in which both stimuli were shown on the screen (**A**). Mice were then trained under a Fixed Ratio-based Effort Discounting (FRED) protocol. The general design was the same as magnitude discrimination training, but the schedule of reinforcement (Fixed Ratio (FR)) assigned to the HR stimulus was varied across each trial block in the sequence FR1, 6, 12 and 18. The order of demand presentation was reversed in the FRED task from ascending to descending effort. Touching the HR stimulus caused the LR stimulus to disappear while the HR stimulus remained present on the screen until High Effort/Large Reward (HE/HR) trial completion. If a mouse stopped responding for 40 s and a HE/HR trial was not completed (scored as a within-trial omission), a new trial started. After trial completion, a tone (500 ms, 3 kHz) was issued, the magazine was illuminated, and animals received the assigned amount of milkshake (HR = 40 μl; LR = 5 μl). After reward collection, a new trial started after 1 s ITI (**B**). After baseline stabilization on FRED, trial length (time between the first HE response and the last one followed by reinforcer delivery in each trial per block) was calculated as an average (seconds) of three consecutive days: 0.250 (FR1); 5.52 ± 0.25 (FR6); 11.85 ± 0.61 (FR12); 19.33 ± 0.61 (FR18). These delays were added after every LE/LR choice in the corresponding block of trials for the haloperidol test. Animals were then re-trained 1 session/day in this task until baseline stabilization (**C**). Delays were checked and re-calculated for the subsequent d-amphetamine experiment: 0.250 (FR1); 4.10 ± 0.18 (FR6); 12.86 ± 2.05 (FR12); 18.9 ± 1.40 (FR18). Bottom panel: Experimental timeline (**a**–**f**).

Pharmacological challenges were delivered using a within-subject design according to a Latin-square dose assignment and separated by a minimum of 3 days during which animals were re-baselined on the behavioral task of interest. Haloperidol and/or d-amphetamine were injected intraperitoneally (IP) 40 and 30 min respectively before testing at a volume of 10 ml/kg. Doses and timing are presented in Figs. 1 and 2 (experimental timelines).

### Apparatus

All experiments were performed in standard mouse touchscreen chambers (Campden Instruments Ltd, Loughborough, UK) [40] illustrated in Figs. 1 and 2.

### Behavioral procedures

#### Habituation and operant pre-training

After animal facility acclimatization, mice were food restricted to maintain 85–90% of free-feeding body weight.

Once weights stabilized, animals were exposed to the reward (strawberry milkshake, Yazoo, Friesland Campina Ltd, Horsham, UK) in their home cages overnight to reduce neophobia. For the next two consecutive days, animals experienced a 20-min habituation to the touchscreen chambers with 200 µl of milkshake in the reward collection magazine with no programmed consequences. Following habituation, animals were pre-trained to learn touch-reward contingencies following an “initial touch” protocol (Supplementary Materials). After pre-training one group of animals was assigned to Experiments 1–3 (*N* = 43) and another to Experiment 4 (*N* = 13) focused on FRED development. See timelines in Figs. 1 and 2a–f.

### Experiments 1–3: Rearing Effort Discounting (RED) task

RED training consisted of three main stages (parameters explained in more detail in Fig. 1):

#### Reward magnitude discrimination training

Animals were trained to discriminate between a High Reward (HR; 40 µl) and a Low Reward (LR; 5 µl) stimulus (Fig. 1A).

#### High effort (HE) training

Animals were trained to touch increasingly higher HR stimuli and the highest Response Height reached was recorded for each animal. The highest Response Height reached by >90% of the animals was selected for the next stages (Fig. 1B).

#### RED task

Animals could choose between the HE stimulus presented at different heights (HE/HR option) or the Low Effort (LE) stimulus associated with the LR that remained in an easily reachable position (LE/LR option) (Fig. 1C).

### Experimental timelines

Experiment 1: after completing training (Fig. 1a–c), one group of animals (Fig. 1) was tested on RED after haloperidol and d-amphetamine administration, allowing behavioral baseline stabilization between tests. Haloperidol was also tested in the “HE training” schedule (Fig. 1d) to assess the ability of drug treated animals to reach the HE stimuli in the absence of a LE option. After a re-baseline on RED, a pre-feeding experiment was carried out to test task reward devaluation sensitivity (Fig. 1e) (pre-feeding protocol and results in Supplementary Materials). Finally animals were presented with RED with descending Response Height order (Fig. 1f). Experiment 3: animals were tested in RED with randomized Response Height. Drug doses and testing order are illustrated in Fig. 1.

### Experiment 4: Fixed-Ratio Effort Discounting (FRED) task with and without equivalent delays

The FRED task consisted of three stages: magnitude discrimination training, in which mice learned to discriminate between a HR and LR option (Fig. 2A), the FRED task (for trial length calculation), in which the effort required to obtain the HR increased throughout the session (from FR1 to FR12) while the effort to get the LR remained stable at FR1 (Fig. 2B), and FRED with equivalent delays, in which the average time between the first HE response and reward delivery was adjusted after the LE response for every block of trials [18, 19] (Fig. 2C).

### Experimental timeline

In Experiment 4, animals went through the three main stages of training (Fig. 2a–c). When behavior was stable in FRED with equivalent delays, mice were tested first with haloperidol and then d-amphetamine. Mice were then re-trained in FRED (with non-equivalent delays) and experienced the same pharmacological manipulations (Fig. 2d). After re-baselining on FRED, mice were tested on FRED under pre-feeding conditions (Fig. 2e) (protocol and results in Supplementary Materials) and then presented with a FRED schedule with descending effort demand (Fig. 2f).

### Statistical analysis

Our primary dependent measures in the RED and FRED tasks (the percentages of High Effort/High Reward (HE/HR) choices and percentage of omissions) were analyzed with two-way repeated measures ANOVAs with Dose and Block of trials as two within-subject factors with the Huynh-Feldt correction applied as determined by Mauchly’s test followed by Sidak post hoc analysis to account for multiple comparisons. The HE/HR was calculated by dividing the number HE choices by the total number of choices (excluding omissions). Delay To Reinforcement (time between the first stimulus touch and reward collection) was analyzed by repeated measures ANOVA or by mixed-effects model to account for missing values.

The percentage of LE/LR choices and secondary measures including reward collection latencies, session duration, and front and rear infrared beam breaks as indexes of horizontal locomotion are presented in Supplementary Results.

## Results

### Haloperidol induced a shift from HE/HR to LE/LR in RED (Experiment 1)

**Fig. 3:**
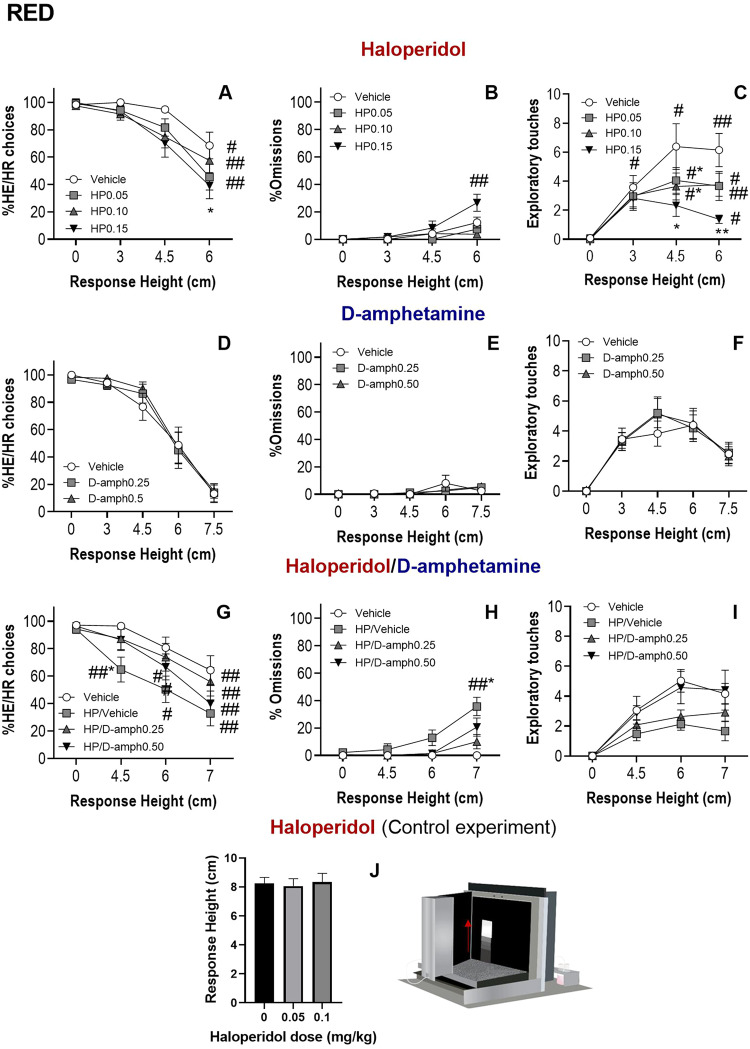
Rearing-Effort Discounting (RED) task: pharmacological characterization. Effects of haloperidol (**A**–**C**). Haloperidol (HP) decreased the percentage of High Effort/High Reward (HE/HR) choices (**A**). Only the highest dose (0.15 mg/kg) increased the percentage of Omissions (**B**). Haloperidol also decreased the number of exploratory touches under the HE stimulus (**C**). Effects of d-amphetamine (**D**–**F**). d-amphetamine did not have effects on any recorded variables at either moderate doses (0.5 and 0.25 mg/kg) (**D**–**F**) or a higher dose (1.0 mg/kg) (Supplementary Results). Effects of haloperidol (0.10 mg/kg) and d-amphetamine co-administration (**G**–**I**). Haloperidol decreased percentage of HE/HR choices (**G**), and increased omissions (**H**). d-amphetamine reversed these effects (**G**, **H**). The same trend was observed with exploratory touches (**I**). Haloperidol did not impair the ability to respond in the task as animals could still reach Response Heights identical to or higher than those used in the main RED task (**J**) *p < 0.01, **p < 0.05 significant difference from vehicle; ^#^*p* < 0.01, ^##^*p* < 0.05 significant difference from trial block 1 (Response Height 0 cm).

ANOVA revealed a significant main effect of haloperidol dose [*F*(3,33) = 5.40, *p* = 0.004], trial block [*F*(1.31,33) = 25.63, *p* < 0.001] and a Dose × Block interaction [*F*(9,99) = 2.58, *p* = 0.01] on percentage of HE/HR choices. The decrease in percentage of HE/HR choices after vehicle treatment was not significant. However, haloperidol at a dose of 0.05 mg/kg significantly decreased HE/HR choices in block 4 (Response Height 6 cm) compared with block 1 (Response Height 0 cm) (*p* = 0.001). Similar effects were observed at 0.10 mg/kg (Response Height 6 cm, *p* = 0.04) and 0.15 mg/kg (Response Height 6 cm, *p* = <0.001). Only the highest haloperidol dose (0.15 mg/kg) was significantly different from vehicle in block 4 (Response Height 6 cm, *p* = 0.03) (Fig. 3A).

Within-subjects repeated measures ANOVA revealed significant effects of dose [*F*(3,33) = 5.29, *p* = 0.004], trial block [*F*(1.47,16.26) = 12.74, *p* = 0.001] and a Dose × Block interaction [*F*(9,99) = 5.11, *p* < 0.001] on percentage of omissions. However, only the highest dose (0.15 mg/kg) significantly increased the percentage of omissions in block 4 (Response Height 6 cm) compared to block 1 (Response Height 0 cm) (*p* = 0.01) (Fig. 3B). The decrease in HE/HR choices after haloperidol and the low percentage of omissions across the different block of trials indicates that animals shifted preferences from the HE/HR option to the LE/LR option (Supplementary Results, Fig. S1A).

ANOVA showed a significant effect of haloperidol [*F*(1.87, 20.60) = 10.78, *p* < 0.001], Response Height [*F*(3,33) = 10.88, *p* = 0.003] and a Dose × Response Height interaction [*F*(3,99) = 3.53, *p* = 0.001] on exploratory touches. Sidak post hoc analysis revealed a significant increase in exploratory touches in blocks 2, 3 and 4 (Response Height 3, 4.5 and 6 cm; *p* = 0.01, *p* = 0.01 and *p* < 0.001, respectively) compared to block 1 (Response Height 0 cm) after vehicle treatment. This effect was also observed at doses of 0.05 mg/kg (Response Height 3, 4.5 and 6 cm*, p* = 0.01, *p* = 0.005 and *p* = 0.02 respectively) and 0.10 mg/kg (*p* = 0.05, *p* = 0.02 and *p* = 0.01, respectively). This effect was also observed with the highest dose of haloperidol (0.15 mg/kg) in block 4 (Response Height 6 cm, *p* = 0.003). Further comparisons revealed that animals administered haloperidol at 0.1 mg/kg made fewer exploratory touches than under vehicle treatment in block 3 (Response Height 4.5 cm) (*p* = 0.02). This effect was also observed in block 3 and 4 (Response Height 4.5 and 6 cm) when animals were administered the highest dose of haloperidol (0.15 mg/kg) (*p* = 0.02 and *p* = 0.01, respectively) (Fig. 3C).

To control for motor impairments that could disrupt the ability to reach the stimuli, Experiment 1 animals were administrated haloperidol and exposed again to the HE training schedule (Fig. 1B). The Response Height reached at the end of the session was recorded. A repeated measures ANOVA did not show a significant effect of haloperidol dose on reaching up behavior when only the HE option was available [*F*(2,22) = 0.29, *p* = 0.75]. Animals were able to reach and respond to stimuli higher than those presented in the RED task after haloperidol, and the level of performance was not different from vehicle (Fig. 1J). The switch in preferences from HE/HR to LE/LR choices in RED observed after haloperidol administration is therefore not due to alterations in animals’ ability to make high rearing responses.

### d-Amphetamine did not show any effect on RED (Experiment 1)

D-amphetamine did not have any effect on percentage of HE/HR choices, omissions or exploratory touches (Fig. 3D–F). The same lack of effect was observed with a higher dose of d-amphetamine (1.0 mg/kg) (Experiment 2), thus confirming the lack of effects of d-amphetamine in the task (Supplementary Results, Fig. S4A–D).

### D-amphetamine reversed the effects of haloperidol in the RED task (Experiment 2)

Having observed the lack of effect of haloperidol on percentage HE/HR choices with Response Height 3 cm (Fig. 3A) and the decline observed between Response Height 4.5 cm and 7.5 cm on HE/HR choice (Fig. 3D), in this experiment the second block of HE/HR trials started with block 4 (4.5 cm) and the highest stimulus was moved down 0.5 cm, to make it easier to reach and the final Response Height increment less drastic (Response Height 7 cm).

These results (Fig. 3G–I) demonstrate RED’s sensitivity to detect the restoring effects of d-amphetamine on effort-based decision making, demonstrating the potential of the RED task for the study of potential therapeutics (Statistics in Supplementary Results).

### Haloperidol decreased HE/HR but did not increase LE/LR choices in FRED with equivalent delays (Experiment 4)

**Fig. 4:**
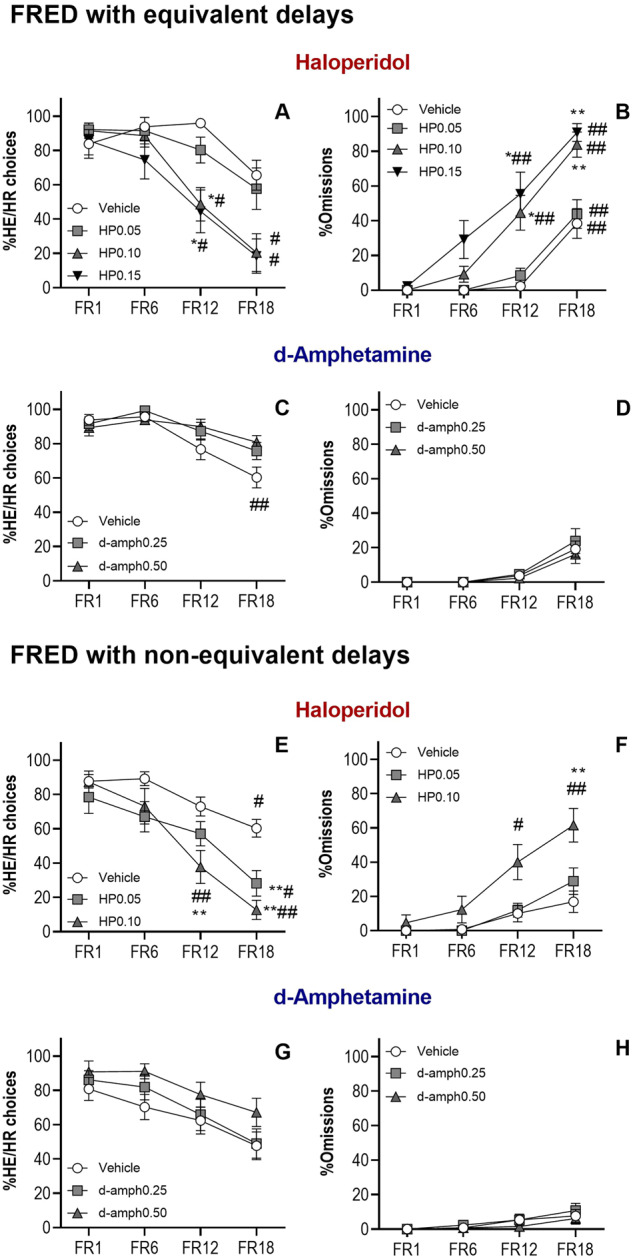
Fixed Ratio-Based Effort Discounting (FRED) task with and without equivalent delays: pharmacological characterization. Fixed Ratio-Based Effort Discounting (FRED) task with equivalent delays: Effects of haloperidol (**A**, **B**). Haloperidol (HP) decreased the percentage of High Effort/High Reward (HE/HR) choices (**A**) and increased the percentage of omissions (**B**). Effects of d-amphetamine (**C**, **D**). d-Amphetamine blunted the effort discounting effect (**C**) and did not have any effect on percentage of omissions (**D**). Fixed Ratio-Based Effort Discounting (FRED) task with non-equivalent delays: effects of haloperidol (**E**, **F**). Haloperidol (HP) decreased the percentage of High Effort/High Reward (HE/HR) choices (**E**). Haloperidol increased percentage of omissions (**F**). Effects of d-amphetamine (**G**, **H**). A non-significant increase in HE/HR choices was observed after d-amphetamine (**G**), and no effect on the percentage of omissions (**H**). *p < 0.05, **p < 0.01 significant differences from vehicle treatment; ^#^*p* < 0.05, ^##^*p* < 0.01 significant differences from vehicle treatment.

An overall significant main effect of haloperidol dose [*F*(3,36) = 5.42, *p* = 0.04], trial block [*F*(3,36) = 21.19, *p* < 0.001] and a Dose × Block interaction on percentage of HE/HR choices [*F*(5.12,61.40,108) = 2.53, *p* = 0.04] was observed. A significant decrease in percentage of HE/HR choices was observed in block 3 (FR12, *p* = 0.02) and block 4 (FR18, *p* = 0.02) after haloperidol at 0.10 mg/kg in comparison with block 1 (FR1). The same pattern of effects was observed after administration of the highest dose (0.15 mg/kg) in block 3 (FR12, *p* = 0.01) and block 4 (FR18, *p* = 0.03). When comparing the effects of haloperidol doses with vehicle in the different blocks, significant differences were observed in block 3 (FR12) in which haloperidol (0.10 and 0.15 mg/kg) significantly decreased the percentage of HE/HR choices in comparison to vehicle (*p* = 0.001 and *p* = 0.01, respectively) (Fig. 4A).

There was a significant main effect of haloperidol [*F*(3,36) = 22.73, *p* < 0.001], trial block [*F*(3,36) = 83.228, *p* < 0.001] and a Dose × Block interaction [*F*(9,108) = 6.03, *p* < 0.001] on percentage of Omissions. When treated with vehicle and haloperidol at 0.05 mg/kg, animals significantly increased omissions in block 4 (FR18 (*p* = 0.004 and *p* = 0.001 respectively) compared with block 1 (FR1). This effect was also observed after haloperidol administration at 0.10 and 0.15 mg/kg in block 3 (FR12) (*p* = 0.005 and *p* < 0.001) and block 4 (FR18) (*p* = 0.001 and *p* = 0.001 respectively) (Fig. 4B).

The behavioral shift from HE/HR to LE/LR was not observed (Supplementary Results, Fig. S5A) due to the increase in omissions induced by haloperidol in this task.

### d-Amphetamine decreased effort discounting in FRED with equivalent delays (Experiment 4)

The opposite pattern of effects to haloperidol was observed on percentage of HE/HR choices and omissions after d-amphetamine treatment in FRED with equivalent delays. Analysis of the choice data revealed significant main effects of d-amphetamine on percentage of HE/HR choices (*F*(2,24) = 2.43, *p* = 0.05), trial block (*F*(3,36) = 20.14, *p* < 0.001) and a significant Dose × Block interaction (*F*(6,72) = 3.06 *p* = 0.01). Sidak post hoc analysis showed a significant decrease in percentage of HE/HR choices in block 4 (FR18) compared with block 1 (FR1) after vehicle treatment (*p* = 0.003). This effect disappeared after administration of 0.25 and 0.5 mg/kg d-amphetamine since percentage HE/HR choices in block 4 (FR18) was not significantly different from block 1 (FR1) (*p* = 0.42 and *p* = 0.63, respectively) (Fig. 4C). Analysis of the percentage of omissions did not reveal a significant effect of d-amphetamine (Fig. 4D). The increase in HE/HR choices was accompanied by a decreasing trend of LE/LR choices (Supplementary Results, Fig. S5B).

### Haloperidol and d-Amphetamine showed opposite effects in FRED with non-equivalent delays (Experiment 4)

Haloperidol significantly decreased the percentage of HE/HR choices and increased omissions (Fig. 4E, F). D-amphetamine did not produce significant effects on this version of FRED, however, the trend of effects was opposite to those observed with haloperidol. Animals administered d-amphetamine increased the percentage of HE/HR choices while without affecting omissions (Fig. 4J–L) this was accompanied by a decreasing trend on the percentage of LE/LR choices (Supplementary Results, Fig. S5D).

### Behavioral measures and manipulations

#### RED but not FRED showed an effort discounting pattern when order of demand was reversed or randomized (Experiments 1–4)

**Fig. 5:**
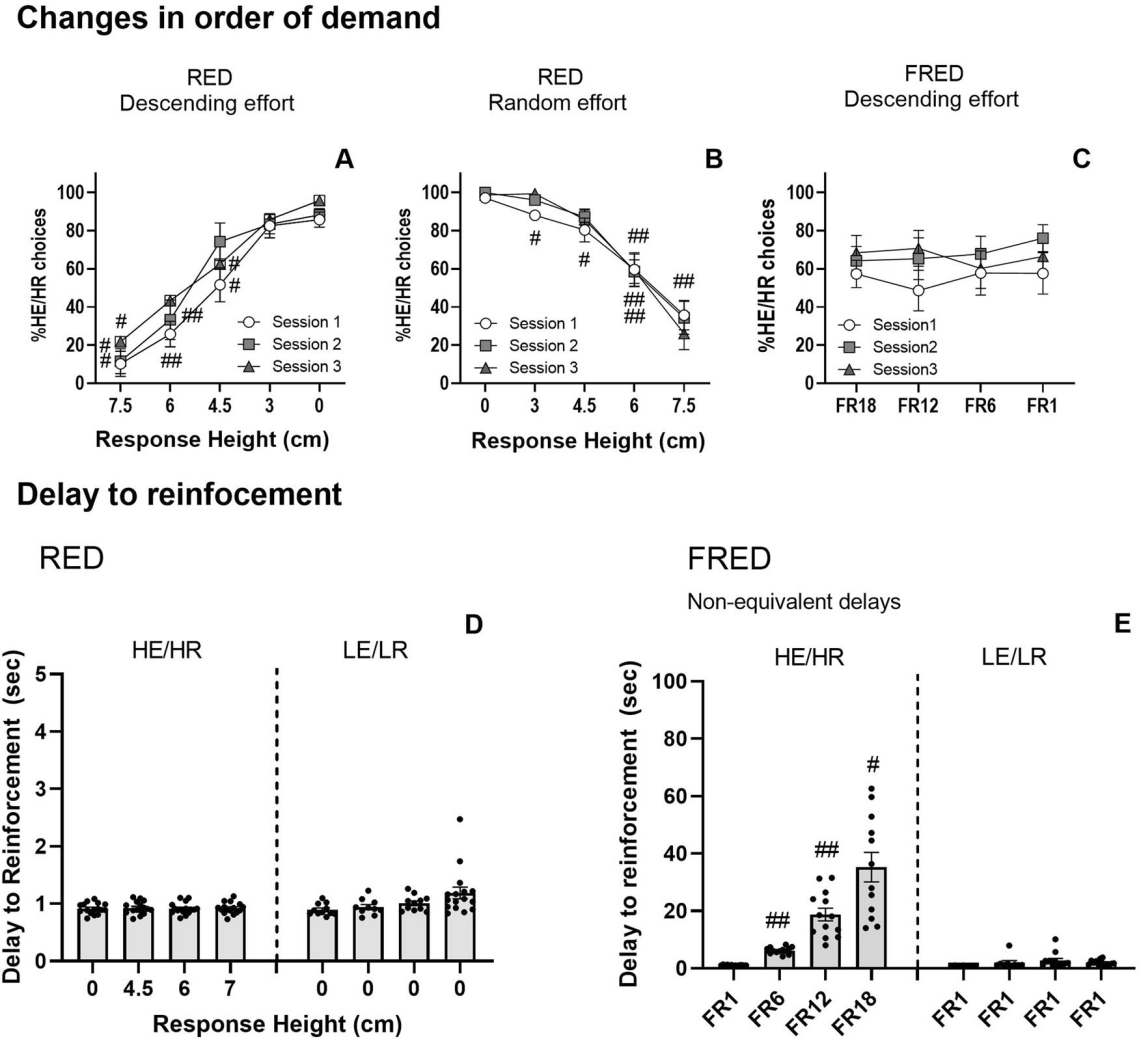
**The Rearing-Effort Discounting (RED) and Fixed Ratio-Based Effort Discounting (FRED) tasks: behavioural measures and manipulations**. The Rearing-Effort Discounting (RED) task with descending and random effort demand (**A**, **B**). The increase in percentage of High Effort/High Reward (HE/HR) choices as the Response Height decreased (**A**). Animals were also sensitive to effort demand when the different Response Heights were displayed in a random order as shown by the decrease in the percentage of HE/HR choices when Response Height was high (**B**). Fixed Ratio-based Effort Discounting task with descending effort demand (**C**). This design was not sensitive to changes in the order of effort demand and animals did not show an effort discounting profile. Delay to reinforcement in RED and FRED (non-equivalent delays task during baseline) (**D**, **E**). Delay to reinforcement did not change with effort requirement (Response Height) in RED and values were very similar for High Effort/High Reward (HE/HR) and (LE/LR) options (**A**). However, delay increased in FRED for the HE/HR option as the fixed ratio (FR) requirement increased. Delays to reinforcement values were different between the HE/HR and LE/LR option as the latter was fixed at FR1 (**B**). ^#^*p* < 0.01, ^##^*p* < 0.05 significant differences from Response Height 0 cm.

RED was sensitive to changes in effort demand when Response Height was changed to a descending order (Fig. 5A) (Experiment 1) or when shown in a random order (Fig. 5B) (Experiment 3). In both cases mice changed their preferences to low effort depending on effort demand (Supplementary Results). This was not the case for FRED when the effort demand was changed to a descending order (Fig. 5C) (Experiment 4), thus highlighting the flexibility offered by RED.

#### Delay to reinforcement was smaller in RED than in FRED for every block of trials

To test the ability of RED to minimize the delays to reinforcement and equalize them between the HE/HR and LE/LR option, the time between the first touch to reward collection was calculated for both FRED and RED using an average of three consecutive training days during baseline (Fig. 5D, E). Changes in delay to reinforcement were not observed across the block of trials in RED for the HE/HR option or for the LE/LR option (*F*(1.59, 23.91) = 0.60, *p* = 0.52 and *F*(1.509, 14.58) = 3.659, *p* = 0.06), respectively) and values were very similar for both options (Fig. 5D). In contrast, in FRED the delay of reinforcement significantly increased as the fixed ratio (FR) requirement increased in the HE/HR option (*F*(1.210, 14.12) = 41.29, *p* < 0.001) but not for the LE/LR option *F*(2.032, 17.61) = 1.493, *p* = 0.25) (Fig. 5E). These findings show that RED does not feature the same potentially confounding delays to reinforcement as are found in FRED and similar tasks.

## Discussion

Robust and reliable effort-based decision-making tests are essential for preclinical research into neuropsychiatric diseases in which decision-making ability is compromised. Currently available tests are limited, however, by factors such as the presence of delayed reinforcement and off-target cognitive demands. In the present study we developed and validated a touchscreen-based effort discounting task for mice, RED, in which these confounds have been minimized. The RED task capitalizes on the flexibility of the touchscreen testing method by using a variable “reaching up” requirement for manipulating cost. We validated RED in several ways, including by comparison with a touchscreen version of conventional fixed-ratio discounting (FRED), behavioral manipulations, pre-feeding, and the use of dopaminergic drugs with well-known profiles on existing decision-making tests.

RED was shown, like other decision-making tests [8, 10, 12, 18, 38], to be sensitive to antagonism of the dopamine system. Haloperidol reduced the preference of mice to work harder to obtain a High Reward in RED, in accordance with previous studies [10, 12, 15]. Haloperidol decreased the percentage of High Effort choices (Fig. 3A) and increased Low Effort (Fig. S1A) choices at all doses without increasing omissions (Fig. 3B). Only the highest dose used (0.15 mg/kg) increased omissions; this dose also significantly affected locomotion (as shown by IR beam breaks, Table S1). The shift from HE/HR choices to LE/LR choices after haloperidol in RED was not due to the effect of this drug on animals’ rearing ability since, when the Low Effort option was not available, animals were able to reach higher Response Heights than the ones used in RED (Fig. 3J). This is also supported by previous studies which have shown that mice can reach a panel located at 6.1 cm high around 100 times after haloperidol (0.1 mg/kg) [12]. The shift from High Effort to Low Effort choices was also observed in the FRED task with non-equivalent delays as it is conventionally run (Fig. 4E–H), but not when delays were adjusted to be equivalent; under these conditions, the haloperidol-induced decrease in HE/HR choices was not accompanied by an increase in LE/LR choices. Instead, a greater increase in omissions was observed, particularly in the latter parts of the session when effort demand and therefore the delay associated with its completion increased (Fig. 4A, B). This finding suggests that haloperidol decreases willingness both to work harder and to wait longer for a reward, in accordance with previous studies using the dopamine receptor antagonist flupenthixol [18].

D-amphetamine did not, however, have an effect in the RED task (Fig. 3D, E). In previous studies, d-amphetamine was shown to increase responding in progressive ratio schedules [40, 41] and to increase HE/HR option choices in conventional fixed-ratio effort discounting tests in rats [18, 37]. The reason for these differing results may be the presence of off-target task demands in the tests used in these studies. This interpretation is consistent with the significant effects of d-amphetamine observed in our touchscreen version of fixed-ratio discounting (FRED) and in preliminary studies carried out in our laboratory using FRED with equivalent delays at 1.0 mg/kg [42].

Although d-amphetamine had no effect in the RED task when administered alone, the sensitivity of RED to d-amphetamine challenge was confirmed by a robust reversal by d-amphetamine of a haloperidol-induced shift in preference from the High Effort to the Low Effort option having only a small effect on the number of omissions (Fig. 3G, H). A trend toward d-amphetamine-induced reversal was also observed on the number of “exploratory touches” under the HE stimulus which can be taken as an index of willingness to exert the optimal effort to reach the target (Fig. 3I). These findings underscore RED’s promise as a tool for testing therapeutic approaches to treating impairments in decision-making.

Comparison of RED with conventional decision-making tests, which require highly demanding fixed- or progressive-ratio schedules (and the associated trial length or delay of reinforcement), or with those with fixed designs, reveals several advantages, discussed below.

In fixed- and progressive-ratio-based decision-making tasks, to make a choice subjects must estimate the effort required to yield reward from one of two identical stimuli. With no cue to indicate effort required, subjects need to calculate, infer, or remember the effort associated with the HE stimuli, an additional cognitive load which may influence performance. That such factors might be a problem was made very clear by the finding that when the order of the different HE/HR options was changed from ascending (i.e., FR1 to FR20) to descending (i.e., FR20 to FR1), HE/HR choices did not increase when the effort decreased, as would be expected in an unconfounded discounting task [18]. We replicated this effect in our touchscreen-based FRED task (Fig. 5C). With RED however, we observed a clear increase in HE/HR choices across the session when the Response Heights were shown in descending order (Fig. 5A). Indeed, the expected response curve was found in RED even when Response Heights were presented in random order (Fig. 5B). These findings indicate that RED minimizes the confounds and carry-over effects that make fixed- (and progressive-) ratio-based decision-making tests inflexible in the way choices can be presented. RED allows animals to make choices on a trial-by-trial basis, based on explicitly and unambiguously presented effort demands.

Another possible confound present in fixed- and progressive-ratio-based decision-making tasks is that the higher the number of responses required, the longer the delay between response initiation and reward. Thus, subjects’ decisions may be based not on effort, but on how long one must wait to receive reward. This difficulty for interpretation is particularly problematic in the present context as it is known that increases in DA transmission with psychostimulants such as d-amphetamine can shorten time perception, [34, 43] and DA antagonism can lengthen it [43]. Perhaps as a result, psychostimulants such as d-amphetamine have been shown to increase tolerance of delays [18] (sometimes interpreted in terms of “waiting impulsivity” [44–47]). Indeed, it has been reported that tolerance to delays in delay-discounting tasks positively correlates with performance in a fixed ratio-based effort discounting task, consistent with a delay discounting component in both types of task [48, 49]. The response requirements in RED do not introduce a delay confound and are symmetrical: both options require a single response, and reward is delivered immediately. This has been shown in Fig. 5D which not only shows symmetrical values for the high effort and low effort responses, but also very small latency values when compared to those present in the final blocks of FRED (Fig. 5E). This may explain why d-amphetamine had no effect on RED, but did affect FRED and other fixed- and progressive-ratio-based designs. Moreover, session duration is shorter in RED than FRED, which is preferable when assessing the acute effects of pharmacological manipulations (see Supplementary Results).

Another methodological limitation in most two-choice fixed-ratio designs is that the Low Effort option becomes unavailable once the animals make the first High Effort choice. Thus, animals must deliver the full complement of touches (or lever presses [18, 19, 49]) required to complete a High Effort trial and obtain the reinforcer, or fail to complete the demand (within-trial omission shown in Supplementary Methods). The touchscreen implementation and nature of the response targets in the RED task, however, allow us to record the ratio of touches under the target stimulus as an index of approaching behavior or willingness to exert the optimal effort to reach the HE stimulus. When the Response Height is high, animals often approach the High Effort stimulus by placing their front paws on the screen and rearing up toward the stimulus in a manner reminiscent of “vicarious trial and error” [50] having the opportunity to shift to the Low Effort option after a first tentative approach. Had the method incorporated the conventional feature of removing the Low Effort option after a single exploratory approach, such mice would be forced to complete the High Effort trial, or omit a response, even though that was not their actual choice. One can see how this arrangement could generate spurious data, a problem minimized in RED.

Another limitation associated with procedures in which subjects are not rewarded until completion of multiple responses is that any manipulation that affects a subject’s ability to complete the full response can yield a pattern that looks like effort-related discounting but is not [51]. For example, some studies have shown that d-amphetamine increases, and DA antagonism decreases, resistance to extinction [52–54]. DA drugs can affect perseveration (e.g., d-amphetamine increasing it and haloperidol decreasing it [22, 54], habit formation [55, 56], and impulsivity [57–59], all of which are related and could affect performance on repetitive-response tasks in a way that is not about effort-based decision making per se. D-amphetamine has been demonstrated to increase lever pressing in Progressive Ratio schedules [40, 58, 59] which are sensitive to the arousing effects of psychostimulants (e.g., methylphenidate) [6]. RED, however, does not feature repetitive responding, thus minimizing such potential confounds.

In rodents, as in humans, repetitive responding can become somewhat inflexible or automatic. This could be a consideration in tasks in which the preferred stimulus or choice is placed repeatedly in a fixed location. Across training, initially goal-directed responses may become automatic and inflexible [55, 60], and any manipulation that affects such a response pattern may appear to alter decision-making, when in fact it may be altering the flexibility of responding. For example, in a two-lever delay discounting task the pattern of responses emitted early in a session became fixed and inflexible following acute d-amphetamine or methylphenidate administration independently of the delay associated with the response [22]. The automatism of responses directed to the High Reward stimuli and carry-over effects are minimized in RED because the stimuli are not always fixed in the same location and subjects make a stimulus-based decision for every Response Height displayed on the screen. This is further evidenced  by using the randomized design (Experiment 3), in which the Responses Heights change trial-by-trial. This could explain the enhancing effect of d-amphetamine on HE/HR lever presses [18] in FRED, but not RED. This problem may also underly the increase in HE/HR responses following d-amphetamine in t-maze-based effort discounting tasks [37].

## Summary and future directions

The present study provides further and novel evidence indicating that currently available tests of effort-based decision-making can be limited by off-target cognitive demands and delayed reinforcement that render data, especially those from highly demanding FR schedules and those from dopaminergic manipulations, difficult to interpret. RED is a new validated touchscreen-based effort discounting task for mice in which these confounds have been minimized. Moreover, the flexibility of RED carried out in touchscreens allows the adaptation of Response Height for the study of animals of different sizes due to strain, age, sex, diet, or other factors. Future research will explore this potential as well as the sensitivity of RED to further pharmacological and other manipulations.

### Supplementary information

Supplementary Materials and Results

RED task with random order of effort demand
