# Supplementary material for: Refining the study of decision-making in animals: differential effects of d-amphetamine and haloperidol in a novel touchscreen-automated Rearing-Effort Discounting (RED) task and the Fixed-Ratio Effort Discounting (FRED) task
Source: Neuropsychopharmacology. 2023 Aug 29;49(2):422–32. doi: 10.1038/s41386-023-01707-z (PMC10724152; doi:10.1038/s41386-023-01707-z)
Supplement: Supplementary file 1 — Supplementary Materials and Results [file 41386_2023_1707_MOESM1_ESM.docx]

Supplementary Materials and Methods and Results

**Refining the study of decision-making in animals: Differential effects of d-amphetamine and haloperidol in a novel touchscreen-automated Rearing-Effort Discounting (RED) task and the Fixed-Ratio Effort Discounting (FRED) task.**

Lopez-Cruz L*^1^ PhD, Phillips BU^1^ PhD, Hailwood JM^1^ PhD, Saksida LM^3^ PhD, Heath CJ^2+^ PhD, Bussey TJ^3+^ PhD.

1. Department of Psychology and MRC/Wellcome Trust Behavioural and Clinical Neuroscience Institute, University of Cambridge, Downing Street, Cambridge, CB2 3EB, UK.

2. School of Life, Health and Chemical Sciences, The Open University, Walton Hall, Milton Keynes, MK7 6AA, UK.

3. Robarts Research Institute & Department of Physiology and Pharmacology, Schulich School of Medicine & Dentistry, Western University, London, ON, 5C1, Canada.

***Corresponding author**: Laura Lopez-Cruz [laura.lopez-cruz@open.ac.uk](mailto:laura.lopez-cruz@open.ac.uk)

Present address: School of Life, Health and Chemical Sciences, The Open University, Walton Hall, Milton Keynes, MK7 6AA, UK.

**+Senior authors**

**MATERIALS AND METHODS**

**Drugs**

Haloperidol (Bio-techno, UK) was dissolved in 0.2% (w/v) tartaric acid and administered 40 min before testing. d-amphetamine sulphate (Sigma-Aldrich, UK) was dissolved in 0.9% (w/v) saline and injected 30 min before testing.

**Housing conditions**

Mice were 8-10 weeks old at the beginning of the experiments and were housed in groups of 4 in conventional cages in a humidity- and temperature-controlled housing room (lights off 07:00, lights on 19:00). Mice were left undisturbed aside from routine husbandry for 7 days following arrival to acclimate before handling commenced.

**Operant pre-training**

‘Initial touch’ protocol

Following habituation to the chambers, all animals were trained to touch the screen. At the beginning of the session, one of two screen locations was illuminated and remained illuminated until either 30 seconds had elapsed (upon which 20 µl of milkshake reward was delivered; 800ms pump time) or the illuminated location was touched (upon which 60 μl of milkshake reward was delivered; 2400ms pump time). If the mouse touched the stimulus location while illuminated, the stimulus was immediately turned off, the tone and milkshake reward issued, and the magazine illuminated. Upon reward collection, the magazine light was turned off and a 5-s inter-trial interval (ITI) followed. Animals were considered successfully trained on this phase once 30 rewards were collected during a session (maximum session time = 60 minutes).

After this training, animals performed the Magnitude discrimination training as explained in the Main Manuscript.

**Pre-feeding protocol**

To test the sensitivity of RED and FRED to reward devaluation animals were pre-fed overnight, specifically with standard laboratory chow which was available *ad libitum* and a bowl of milkshake that was placed in their home cages the night before the test. Performance under pre-feeding was compared with baseline performance measured the day before.

**Statistical Analysis**

The percentage of Low Effort/Low Reward (LE/LR) choices was calculated and analysed in the same way as the percentage of High Effort/High Reward (HE/HR) choices presented in the Main Manuscript in both RED and FRED.

Pre-feeding experiments were analysed by repeated measures ANOVA followed by Sidak *post hoc* analysis to account for multiple comparisons.

In the FRED task, the percentages of trials completed and within-trial omissions were also analysed with the same statistics used above.

Post-reinforcement Pause, Reward Collection Latencies, Session duration and infrared (IR) rear and front beam breaks were analysed for all tasks with one-way repeated-measures ANOVA followed by Bonferroni *post hoc* to account for multiple comparisons.

Analyses were conducted using SPSS 20.0 (IBMCorp. Armonk, NY, USA) and graphs drawn with GraphPad prism 8.4.3 (GraphPad Software, San Diego, California USA). Data are presented as mean (±SEM). p<0.05 was considered statistically significant.

**High Effort training and Response Height selection**

Once an animal learned to discriminate between stimuli (reward magnitudes) and showed a preference of ≥80% for the high reward (HR) for at least three consecutive sessions, animals were trained on rearing (reaching up) behaviour (HE training, Fig. 1B Main Manuscript).

100% of the Experiment 1 animals were able to reach stimulus 4 (Response Height 4.5 cm), 91.67% of the mice reached stimulus 5 (Response Height 6 cm), 56% reached stimulus 6 (Response Height 7.5 cm) and 16.67% reached stimulus 7 (Response Height 9 cm). Based on this information, Response Heights for stimuli 1, 3, 4 and 5 were selected as the HE/HR option for testing the effects of haloperidol in RED in the first study (Fig. 3A-C**,** Main Manuscript). The Response Heights were double checked before the d-amphetamine experiment to test for possible effects of training on the “HE training schedule”, and the percentages were very similar (Response Height 4.5 cm: 100%, 6 cm: 91.67%, 7.5 cm: 54.1%, 9 cm: 11.3%, 10.5 cm: 0%). After considering a possible ceiling effect and to give an opportunity to those animals able to reach stimulus 6 during the HE training to reach higher stimuli, another stimulus (Response Height 7.5cm) was added for the d-amphetamine test (Fig. 3D-F-L, Main Manuscript).

This procedure was repeated for Experiments 2, and very similar results were obtained. However, the last Response Height was adjusted (moved down 0.5 cm), as explained in the Main Manuscript.

**RESULTS**

**Reward Magnitude Discrimination training in RED and FRED**

**Daily magnitude discrimination training in RED (Experiment 1).** Repeated measures ANOVA showed a significant effect of session on the Percentage of HR choices [F(7,105)=16.46, p<0.001]. Bonferroni *post hoc* analysis showed a significant increase in HR (40 µ) preference in sessions 3-8 (p<0.01) (Fig. 1a). An overall effect of session on percentage of omissions was found [F(7,105)=5.46, p=0.000]. Omissions significantly decreased after the first session of discrimination (mean by session (S): S1: 5.20±1.55; S2: 0.45±0.28; S3: 0.21±0.21; S4: 0.83±0.37; S5: 1.04±0.66; S6: 0.83±0.37; S7: 1.25±0.42; S8: 0.83±0.37; p<0.001), thus suggesting that animals learned to discriminate between both stimuli and could complete the trials without experiencing satiation. The same training procedures and similar results were observed in **Experiments 2** and **3** (data not shown).

**Daily magnitude discrimination training in FRED (Experiment 4).**   Repeated measures ANOVA revealed an overall effect of session on the percentage of HR (40 µl) choices [F(13, 156)=10.54, *p<*0.001]. Bonferroni *post hoc* analysis showed an overall significant effect on animal HR preference in sessions 8-14 when compared to session 1 (p<0.001) (Fig. 2a). There was not a significant effect of session on percentage of omissions [F(13,156)=1.00, p=0.45] (mean±SEM per session (S): S1: 1.79±1.04, S2: 2.30±0.69; S3: 0.00±0.00, S4: 2.30±2.30, S5: 0.00±0.00, S6: 0.00±0.00, S7: 0.26±0.26, S8: 0.51±0.35, S9: 0.00±0.00, S10: 1.79±1.79, S11: 0.77±0.55, S12: 0.00±0.00: S13: 0.76±0.41, S14: 0.26±0.24).

***Impact of Haloperidol on LE/LR choices in RED (Experiment 1)***

Haloperidol had an overall effect on percentage of LE/LR choices in RED, indicated by a significant main effect of dose [F(3,33)=4.80, *p=*0.007], trial block (defined by the Response Height of the HE/HR stimulus presented in the trial) [F(1.38, 5.99)=26.59, *p<0.001*] and a Dose x Block interaction [F(5.99,99)=2.17, *p=*0.05]. *Post hoc* analysis indicated a significant increase in the percentage of LE/LR choices after vehicle treatment in block 4 (Response Height 6cm) compared to block 1 (Response Height 0cm) (*p=* 0.04) (Fig. S1AB). Similar effects were observed after haloperidol administration at 0.05 mg/kg (Response Height 6cm, *p<*0.001), 0.10 mg/kg (Response Height 3cm, *p=*0.70; 4.5cm *p=*0.21; 6cm, *p=*0.04) and 0.15 mg/kg (Response Height 3cm, *p=*0.29; 4.5cm, *p=*0.11; 6cm, *p<*0.001). When different haloperidol doses were compared with vehicle treatment significant differences were observed only when animals were presented with Response Height 6 cm (block 4) after administration of the higher dose of haloperidol (0.15 mg/kg) (*p=*0.04) (Fig.S1A)

*
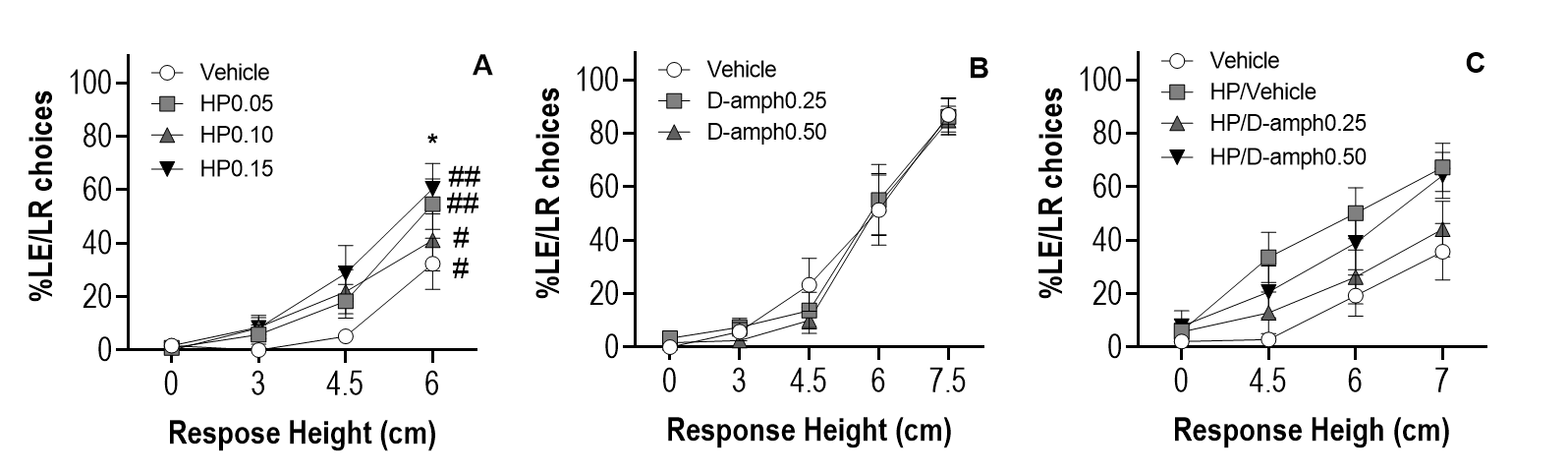
*

**Fig. S1 Effects of haloperidol, d-amphetamine and co-administration on LE/LR choice in RED as a function of trial type (defined by HE/HR stimulus response height) ;** effects of haloperidol (A), d-amphetamine (B) and haloperidol-d-amphetamine co-administration on the Percentage of Low Effort/Low Reward (LE/LR) choices in RED.

***Effect of haloperidol on* *additional performance measures in RED***

**
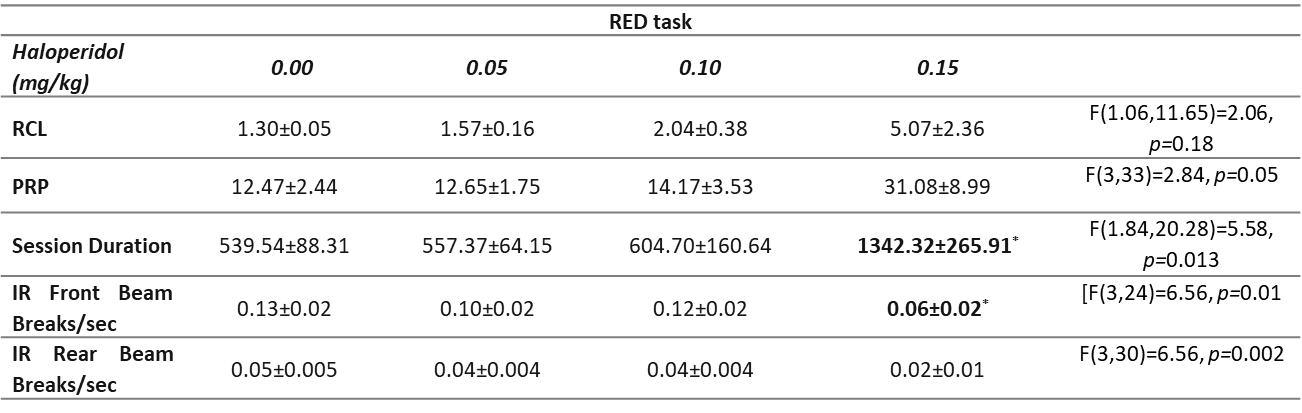
**

**Table S1. Summary of additional performance measures in RED under haloperidol treatment.** Haloperidol did not have effects on Reward Collection Latency (RCL) and Post-Reinforcement Pause (PRP), however the highest dose (0.15 mg/kg) significantly increased Session Duration and decreased IR Front and Rear Beam Breaks per second. *p<0.05 significant differences compared with vehicle.

***d-amphetamine at moderate doses (0.25 and 0.50 mg/kg) did not show any effect on RED***

d-amphetamine did not produce any effect in the RED task. Analysis of the percentage of HE/HR choices did not show a significant effect of d-amphetamine dose [F(2,22)=0.58 ,*p=*0.57] nor a Dose x Block interaction [F(8,88)=0.94, *p=*0.49]. However, a significant effect of trial block was found [F(1.70,18.75)=43.02,*p<*0.001] (Fig. 3D, Main Manuscript).

d-amphetamine did not have an effect on Percentage of Omissions [F(2,22)=0.52, *p=*0.60] and there was no significant Dose x Block interaction [F(8,88)=1.06, *p=*0.09]. Only an effect of trial block was observed [F(2.41,26.41)=3.55, *p=*0.04] (Fig. 3E, Main Manuscript).

Within subjects repeated measures ANOVA showed a significant effect of d-amphetamine dose on exploratory touches [F(2,22)=4.07, *p=*0.001] and Response Height [F(2.81,44)=8.20, *p<*0.001] but a non-significant Dose x Response Height interaction [F(5.50,60.46)=0.59, *p=*0.72] (Fig. 3F, Main Manuscript).

Within-subjects repeated measures ANOVA did not indicate significant effects of d-amphetamine on the percentage of LE/LR choices. A significant effect of trial block was observed [F(1.51,18.75)=43.02, *p<*0.001], but not a significant main effect of Dose [F(2,22)=0.59, *p=*0.56] or a Dose x Block interaction [F(8,88)=0.94, *p=*0.49]. (Fig S1B).

***Effect of d-amphetamine on* *additional performance measures in RED*** (Table S1)

***
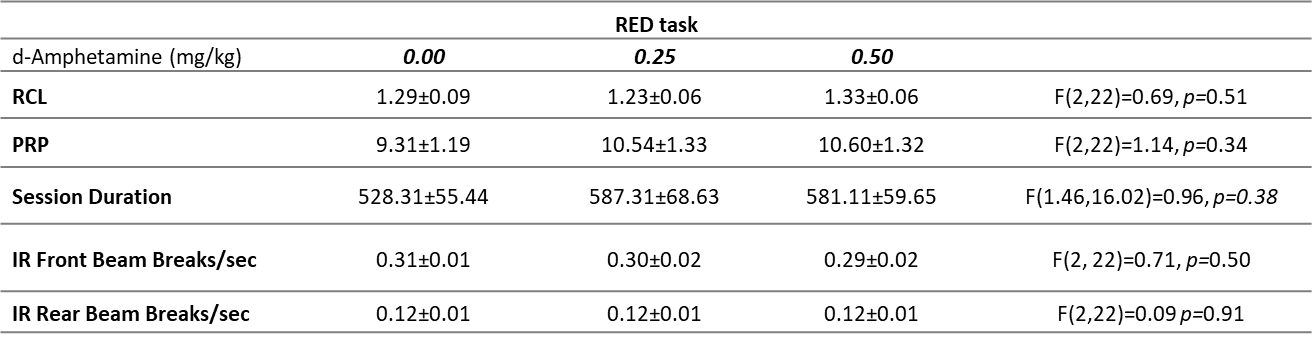
***

**Table S2**. **Summary of additional performance measures in RED under d-amphetamine treatment**. d-amphetamine did not produce any effects on RCL, PRP, Session Duration, IR Front and Rear Beam Breaks per second in RED.

***Impact of pre-feeding on RED***

Repeated measures ANOVA showed a significant main effect of feeding condition [F(1,11)=35.20, *p<*0.001] and Response Height [F(4,44)=40.47 *p<*0.001], but no significant Response Height x feeding condition interaction [F(4,44)=2.29 *p=*0.08] on the percentage of HE/HR choices made in RED (Fig. S3A).

Analysis of the percentage of LE/LR choices did not reveal a main effect of feeding condition [F(1,11)=4.22, *p=*0.06] but a significant main effect of Response Height [F(4,44)=114.90, *p<*0.001] and a significant Response Height x feeding condition interaction [F(2.78,30.53)=7.34, *p=*0.001] were observed. Sidak *post hoc* analysis showed a significant increase in the percentage of LE/LR choices under baseline conditions (mild food restriction) when stimuli 5 (Response Height 6 cm) and 6 (Response Height 7.5 cm) were presented (*p=*0.04 and *p<*0.001). However, when animals were pre-fed, this was only significant when stimulus 3 (Response Height 3 cm) was presented (*p=*0.01).

When animals were pre-fed, they significantly increased the percentage of LE/LR choices, relative to when mildly food restricted. Significant differences in the percentage of LE/LR choices were found between the two feeding conditions in trial blocks 1-3 (Response Height 0cm, *p=*0.01; Response Height 1.5 cm, *p=*0.003; Response Height 3 cm, *p<*0.001). These differences disappeared when the HE/HR option was increased to Response Height 6 and 7.5 cm (block 4 and 5) (Fig. S3B).

An overall effect of Response Height [F(4,44)=12.44, *p<*0.001], feeding condition [F(1,11)=19.78, *p<*0.001], and a Response Height x Feeding condition interaction [F(4,44)=6.29 *p<*0.001] were also observed on percentage of omissions. Under baseline conditions, omissions did not significantly change across trial blocks. However, when animals were pre-fed, the percentage of omissions significantly increased when the HE/HR option was presented at Response Height 7.5 cm (*p=*0.01). Pre-fed animals omitted significantly more than under baseline (mild food restriction) when Response Heights 3cm - 7.5cm were presented (Response Height 3 cm *p=*0.045, Response Height 4.5 cm *p=*0.01 and Response Height 7.5cm *p<*0.001) (Fig. S3C).

**Fig. S3.** **Effects of pre-feeding on RED performance across trial types (defined by HE/HR stimulus response height)**. Pre-feeding increased the overall percentage of High Effort/High Reward (HE/HR) choices (**A)**, the percentage of Low Effort/Low Reward (LE/LR) choices **(B)** and the percentage of omissions (**C)**. **p<0.01*p<0.05 significant differences from baseline. ##p<0.01 significant effect from Response Height 0cm trials (block 1).

***Impact of higher doses of d-*amphetamine (1.0 mg/kg) *on RED* (Experiment 2).**

D-amphetamine did not show any effect on RED at high doses. Repeated measures ANOVA did not indicate an effect of d-amphetamine on the percentage of HE/HR choices [F(2,30)= 0.29, *p=*0.75] and although it revealed an effect of Response Height [F(2.93,7.96)=103.45, *p<*0.001], the d-amphetamine Dose x Response Height interaction was not significant [F(2,120)= 1.22, *p=*0.29] (Fig.S4A).

The same pattern of effects was observed on the percentage of LE/LR choices. Only an effect of Response Height was observed on this variable [F(2.62,39.26)=63.85, *p<*0.001] but not of d-amphetamine dose [F(2,30)= 0.07, *p=*0.93] or a Dose x Response Height interaction [F(2.42,36.37)= 1.43, *p=*0.25] (Fig.S4B).

There was a significant effect of Response Height on percentage of omissions [F(1.29, 19.43)=10.03, p=0.003]. However, there was not a significant effect of d-amphetamine dose [F(2,30)=0.05 p=0.95] or a Dose x Response Height interaction [F(56.93, 132.24)= 043, *p=*0.76] (Fig.S4C).

Repeated measures ANOVA showed a significant effect of d-amphetamine dose on exploratory touches [F(2,30)= 6.11, *p=*0.006] and Response Height [F(2.53,37.93)=10.29, *p<*0.001] but a non-significant Dose x Response Height interaction [F(5.04,75.60)=1.41, *p=*0.20]. Although post-hoc analysis was not performed, a decreasing trend on exploratory touches was observed in trial blocks 3 and 4 (Response Height 4.5 and 6 cm) (Fig.S4D).

**Fig. S4. Effects of higher dose d-amphetamine on RED**. d-Amphetamine did not have effects on any recorded variables (A-D)

***Effects of higher dose d-amphetamine on* *additional performance measures in RED*** (Table S3)

**
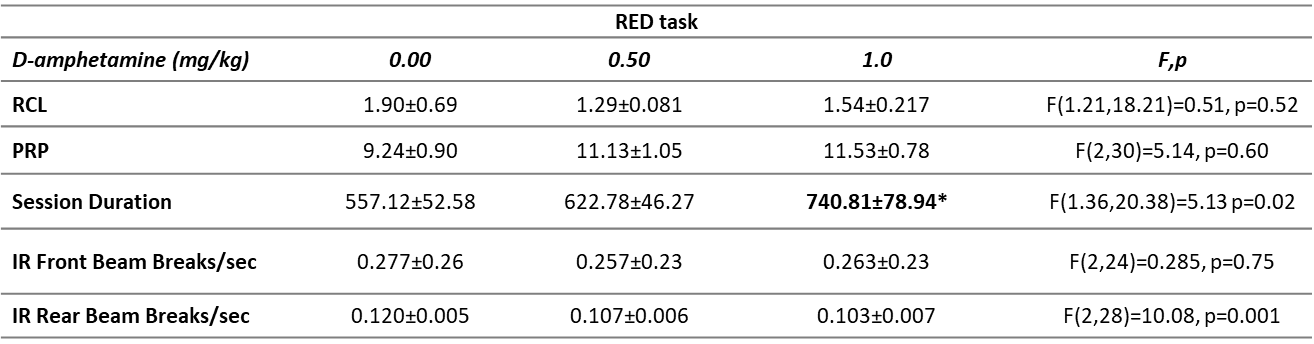
**

**Table S3**. **Summary of additional performance measures in RED under higher dose d-amphetamine treatment**. d-amphetamine did not have any effect on RCL, PRP, Infrared (IR) Front and IR Beam Breaks per second but increased Session Duration at the highest dose used (1.0 mg/kg).

***D-amphetamine reversed the effects of haloperidol in RED***

Analysis revealed a significant main effect of treatment [F(1.97,33.94)=6.57 *p=*0.003], Response Height [F(1.70, 25.48=31.04, *p<*0.001] and a Treatment x Response Height interaction [F(7.20,108.06)=2.26, *p=*0.02] on the percentage of HE/HR choices in RED. After Vehicle-Vehicle (Veh/Veh) administration, animals significantly decreased the percentage of HE/HR choices in block 4 (Response Height 7cm, *p=*0.009). This effect was also observed in blocks 2, 3 and 4 (Response Height 4.5, 6 and 7 cm) when animals were administered Veh/Haloperidol (HP) 0.10 mg/kg (*p*=0.005, *p<*0.001 and *p<*0.001, respectively). A significant decrease in HE/HR choices was observed only in block 4 (Response Height 7cm) (*p=*0.005) when HP/d-Amphetamine (0.25 mg/Kg) was co-administered (Fig. 3G, Main Manuscript). *Post hoc* analysis between treatment groups revealed significantly lower HE/HR choices when animals were co-administered Veh/HP (0.10 mg/kg) compared with Veh/Veh treatment only in block 2 (Response Height 4.5cm, *p=*0.01).

A significant effect of treatment [F(1.71,25.63)=7.64, *p=*0.004], Response Height [F(1.51,45)=20.26, *p<0.001*] and a Treatment x Response Height interaction [F(9,117)=6.22, *p=*0.00] were observed on the percentage of omissions. Significant differences were observed between HP/Veh compared with Veh/Veh treatment in the last block of trials (Response Height 6.5 cm; *p=*0.02). The increase in the percentage of omissions induced by haloperidol was reversed by the co-administration of d-amphetamine at 0.025 mg/kg (*p=*0.39) (Fig. 3H, Main Manuscript). There was a significant increase in the percentage of omissions in the last trial block (Response Height 7 cm) compared to block 1 (Response Height 0cm) when animals received HP/Veh treatment (*p=*0.001). This increase was not observed when animals were co-administered d-amphetamine at 0.25 mg/kg (*p=*0.43) and 0.50 mg/kg (*p=*0.05).

Analysis of the ratio of exploratory touches revealed a significant effect of treatment [F(3,45)=5.04, *p=*0.004] and Response Height [F(3,45)=20.17, *p<*0.001], but a non-significant Treatment x Response Height interaction [F(9,135)=1.56, *p=*0.13]. Although the effect was not significant, haloperidol decreased the number of exploratory touches, and the co-administration of d-amphetamine (0.5 mg/kg) reversed this effect (Fig. 3I, Main Manuscript).

The pattern of results observed on the percentage of LE/LR choices was a mirror image of the effects observed on the percentage of HE/HR choices (Fig. S1C). Animals treated with HP/Veh showed higher LE/LR choices compared with animals treated with Veh/Veh. When animals received haloperidol co-administered with d-amphetamine at 0.25 mg/kg, they showed a similar pattern to Veh/Veh treatment in all the Response Heights presented. However, although the main effects of treatment [F(2.52,37.86)=6.00, *p=*0.003] and Response Height [F(1.68,25.23)=31.23, p<0.001] were significant on this variable, the Treatment x Response Height interaction was not [F(7.43,111.52=1.83, *p=*0.08].

***Effect of haloperidol / d-amphetamine co-administration on* *additional performance measures in RED*** (Table S4)

**
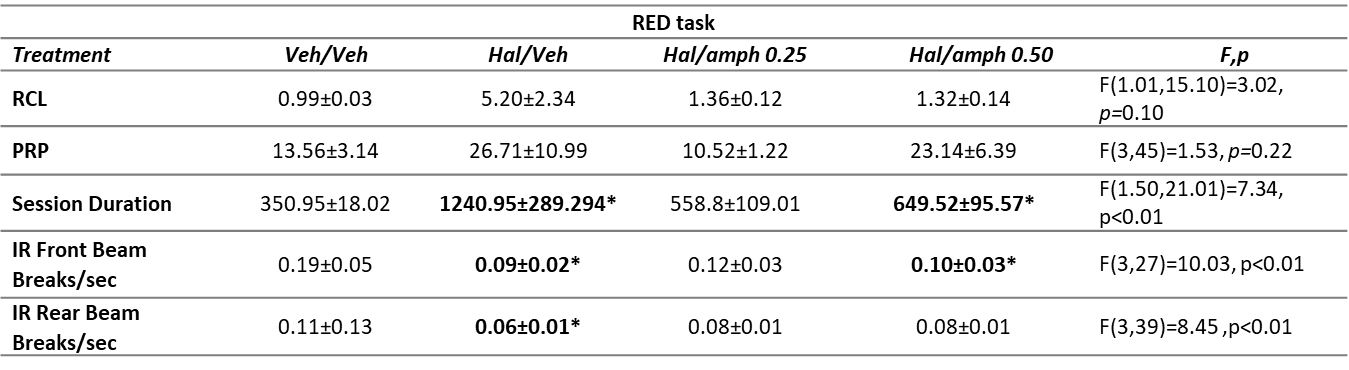
**

**Table S4**. **Summary of additional performance measures in RED under haloperidol / d-amphetamine co-administration**. Haloperidol administration caused increasing trends in RCL and PRP; this trend was not observed with d-amphetamine co-administration. Haloperidol (Hal/Veh) significantly increased Session Duration and this effect was reversed by d-amphetamine (0.25 mg/kg) co-administration. The highest dose of d-amphetamine (0.50 mg/kg) did not reverse the increase in Session Duration induced by haloperidol but did reduce it by approximately 50%. Haloperidol reduced the Infra-Red (IR) Front Beam Break Rate, and this effect was not observed with haloperidol and d-amphetamine (0.50 mg/kg) co-administration. Haloperidol also decreased the IR Rear Beam Break Rate and this was also not observed with haloperidol and d-amphetamine co-administration. *p<0.05 significant difference compared to vehicle.

**Experiment 4**

***Impact of haloperidol on the percentage of LE/LR choices in FRED with equivalent delays***

Analysis of the percentage of LE/LR choices in FRED with equivalent delays did not show an overall effect of haloperidol dose [F(3,36)=1.30, *p=*0.29] or trial block [F(2.02,24.22)=2.79, *p=*0.08]. However a significant Dose x Block interaction was observed [F(9,108)=4.98, *p=*0.001]. Sidak *post hoc* analysis revealed a significant increase only on the percentage of LE/LR choices in block 3 (FR12) after haloperidol at 0.10 mg/kg compared with block 1 (FR1, *p=*0.01). This dose also significantly increased LE/LR choices in this block compared with vehicle (*p=*0.01) (Fig. S5A).


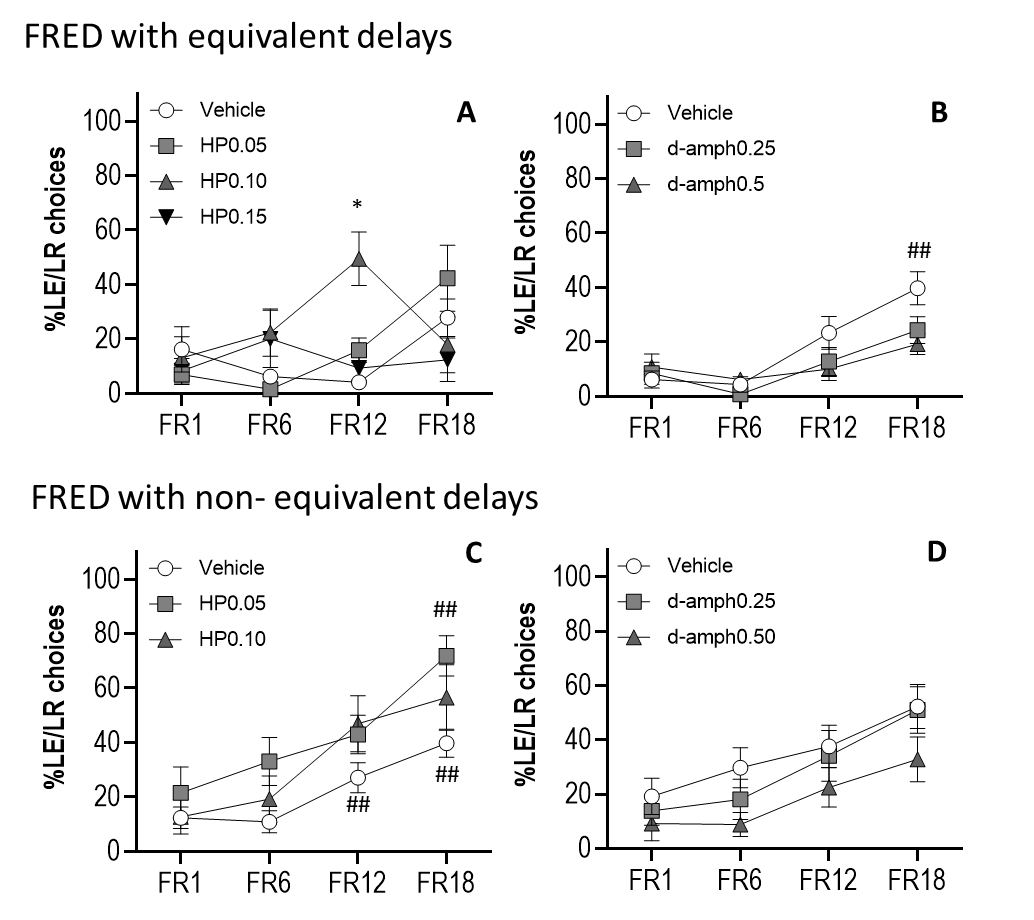


**Fig S5. Effects of haloperidol and d-amphetamine on the percentage of LE/LR choices in FRED across trial types (defined by HE/HR response requirement)** **:** effects of haloperidol (**A**) and d-amphetamine (**B**) on Low Effort/Low Reward (LE/LR) choices in FRED with equivalent delays. **E**ffects of haloperidol (**C**) and d-amphetamine (**D**) on Low Effort/Low Reward (LE/LR) choices in FRED with non-equivalent delays.

**Impact of haloperidol on within-trial omissions in FRED with equivalent delays** (Table S5)

A within-trial omission was recorded in FRED when the animal did not respond to the stimulus within 40 seconds after the HE choice was made.


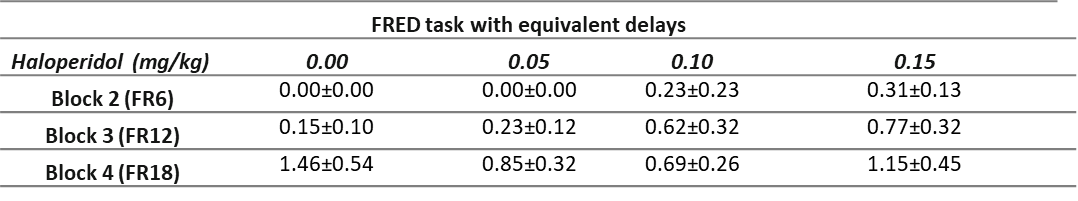


**Table S5. Mean number of within-trial omissions recorded in FRED with equivalent delays under haloperidol treatment as a function of dose and HE/HR response requirement.**

***Effect of haloperidol on* *additional performance measures in FRED with equivalent delays***

**
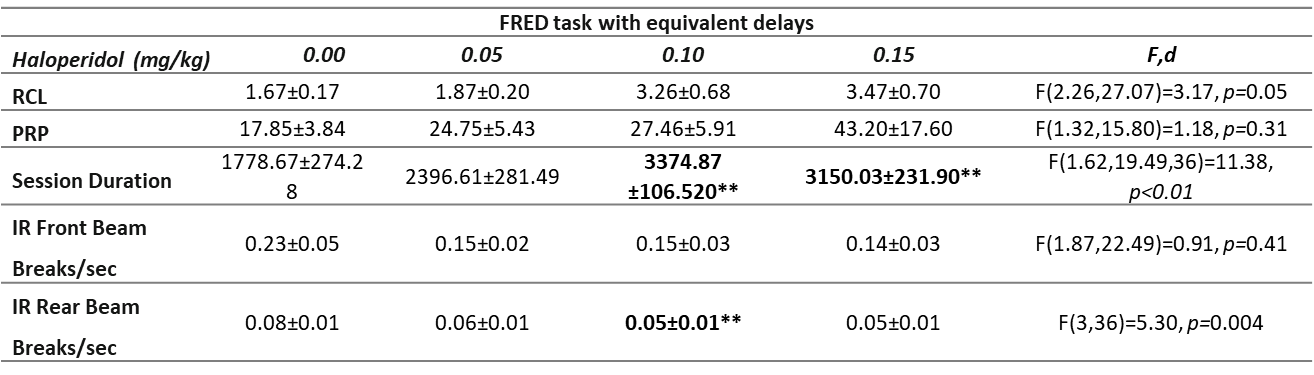
**

**Table S6. Summary of additional performance measures in FRED with equivalent delays under haloperidol treatment.** Haloperidol significantly increased Session Duration, and there was a trend toward increased Reward Collection Latency (RCL) and Post-Reinforcement Pause (PRP). Haloperidol significantly decreased the IR Rear Beam Break Rate but had no significant effect on IR Front Beam Break Rate**.** **p<0.01 significant difference compared to vehicle

***Impact of d-amphetamine on the percentage of LE/LR choices in FRED with equivalent delays***

Significant main effects of dose (F(2,24)=5.03, *p=*0.02), trial block (F(3,36)=19.25, *p<*0.001) and a significant Dose x Block interaction (F(6,72)=3.87, *p=*0.002) were observed on the percentage of LE/LR choices. The percentage of LE/LR choices in block 4 (FR18) significantly increased compared to block 1 (FR1) (*p=*0.01) after vehicle treatment. This effect was also observed after d-amphetamine administration at 0.25 mg/kg (*p=*0.04) but not after the higher dose used (0.5 mg/kg; *p=*0.07) (Fig. S5B).

**Impact of d-amphetamine on within-trial omissions in FRED with equivalent delays**

d-amphetamine dose did not have an overall effect on the number of within trial omissions [F(2,24)=3.20, *p=*0.06], and although there was an effect of trial block on this variable [F(2.34,28.04)=10.32, *p<*0.001], the Dose x Block interaction was not significant [F(6,72)=1.54, *p=*0.18] (Table S7).

**
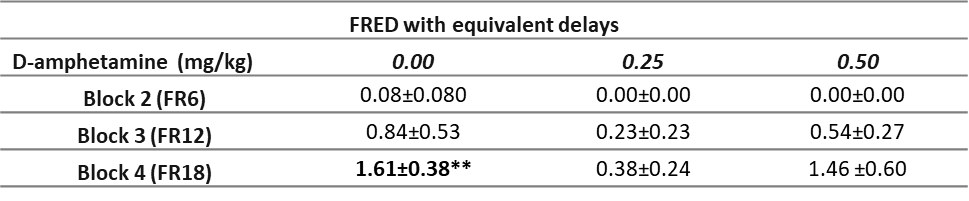
**

**Table S7*.* Mean number of within-trial omissions recorded in FRED with equivalent delays under d-amphetamine treatment as a function of dose and HE/HR response requirement.** Within-trial omissions significantly increased in the control group across blocks. However, this increase was not significant after d-amphetamine administration. **p<0.01 Within-trial omissions in Block 1 is always 0 as the effort requirement was FR1.

***Effect of d-amphetamine on* *additional performance measures in FRED with equivalent delays***

**
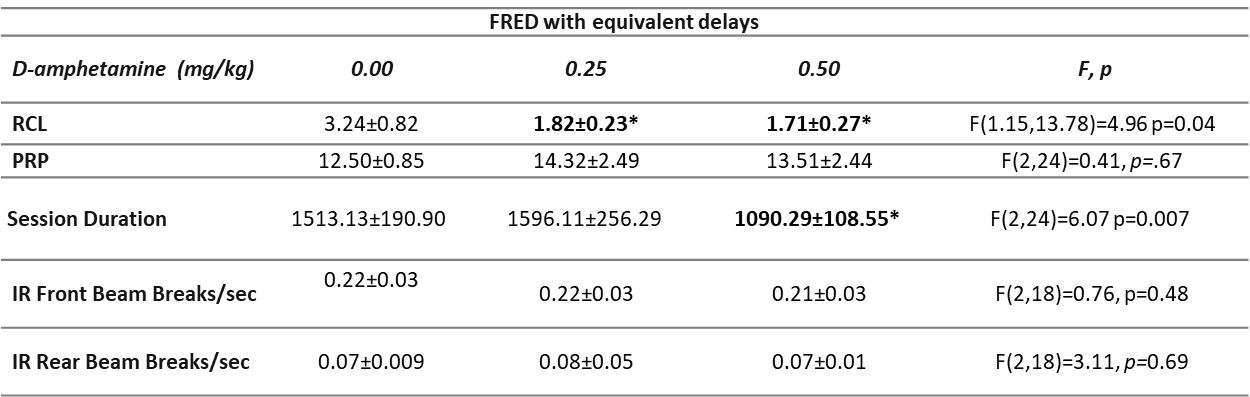
**

**Table S8*.* Summary of additional performance measures in FRED with equivalent delays under d-amphetamine treatment.** D-amphetamine significantly decreased Reward Collection Latency (RCL) and Session Duration. *p<0.05 significantly different to vehicle treatment.

***Opposing effects of haloperidol and d-amphetamine in FRED with non-equivalent delays***

**Impact of haloperidol on FRED with non-equivalent delays**

Analysis of the percentage of HE/HR choices in FRED with non-equivalent delays showed a significant main effect of haloperidol dose [F(2,24)=6.82, *p=*0.05], trial block [F(3,36)=37.07, *p<*0.001] and a Dose x Block interaction [F(6,72)=5.31, *p=*0.003]. Sidak *post hoc* analysis revealed the percentage of choices in block 4 (FR18) was significantly lower than block 1 (FR1) (*p=*0.02). The same effect was observed after haloperidol at 0.05 mg/kg (*p=*0.012). This decrement was also observed in block 3 (FR12, *p=*0.003) and 4 (FR18, *p<*0.001) after the highest dose of haloperidol (0.15 mg/kg).

Compared with vehicle, the percentage of HE/HR choices was significantly lower after haloperidol at 0.10 mg/kg in block 3 (FR12, *p=*0.02). In block 4 (FR18), both doses of haloperidol (0.05 and 0.10 mg/kg) significantly decreased the percentage of HE/HR choices compared with vehicle (*p=*0.003 and *p=*0.003, respectively) (Fig. 4E, Main Manuscript).

An overall effect of haloperidol [F(1.41,24)=9.45, *p=*0.004], trial block [F(3,36)=21.20, *p<*0.001] and a Dose x Block interaction [F(4.81,57.75)=5.20, *p<*0.001] was observed on the percentage of omissions. A significant increase in omissions was observed in block 3 (FR12) and 4 (FR18) after haloperidol (0.1 mg/kg) compared to block 1 (FR1) (*p=*0.05 and *p*<.0012, respectively).

When compared to vehicle, the increase in omissions was only significant in block 4 (FR18) after haloperidol at 0.10 mg/kg (*p=*0.004) (Fig. 4F, Main Manuscript). The increased percentage of omissions and the fact that the decrease in the percentage of HE/HR choices induced by haloperidol was not followed by an increase in LE/LR choices suggests that delay of reinforcement may be also influencing decision making under haloperidol treatment in this task.

Unlike in FRED with equivalent delays, a haloperidol-induced shift from HE/HR to LE/LR choices was observed in this version of the task. However, although within-subjects repeated measures ANOVA revealed a significant main effect of haloperidol [F(2,24)=3.88, *p=*0.04] and trial block [F(3,36)=17.16, *p<*0.001], a significant Dose x Block interaction was not found [F(6,72)=0.99, *p=*0.44] (Fig. S5C), thus further analyses were not performed.

**Impact of haloperidol on within-trial omissions in FRED with non-equivalent delays** (Table S9)

**
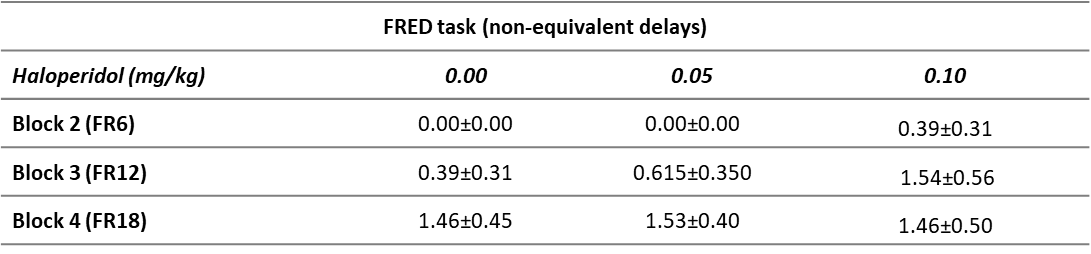
**

**Table S9.**  **Mean number of within-trial omissions recorded in FRED with non-equivalent delays under haloperidol treatment as a function of dose and HE/HR response requirement.**

***Effect of haloperidol on* *additional performance measures in FRED with non-equivalent delays*** (Table S10)
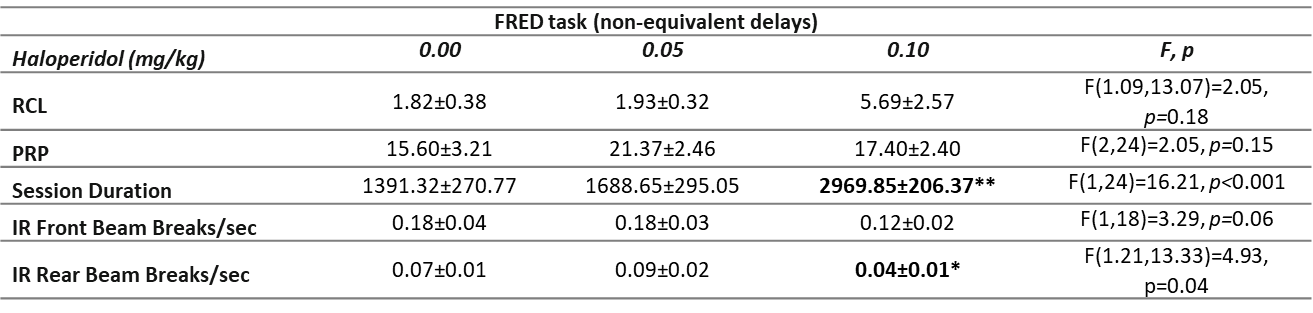
**Table S10.** **Summary of additional performance measures in FRED with non-equivalent delays under haloperidol treatment.** Haloperidol significantly increased Session Duration and decreased Infrared (IR) Rear Beam Break Rate. Significant differences to vehicle were not observed in any of the other variables. (RCL: Reward Collection Latency; PRP: Post-Reinforcement Pause) **p<0.01 *p<0.05 significantly different to vehicle treatment

**Impact of d-amphetamine on FRED with non-equivalent delays**

Analysis of the percentage of omissions revealed a significant effect of trial block (F(1.24,14.92)=4.30, p=0.05) but did not reveal a significant effect of d-amphetamine (F(2,24)=1.92, p=0.17) or a Dose x Block interaction (F(4.28,51.41)=0.82, p=0.52) (Fig. 4H, Main Manuscript).

Significant main effects of dose (F(2,24)=6.93, *p=*0.004) and trial block (F(3,36)=20.95, *p<*0.001) were observed on the percentage of LE/LR choices. However there was not a significant Dose x Block interaction (F(6,72)=0.97, *p=0.45*) (Fig. S5D).

**Impact of d-amphetamine on within-trial omissions in FRED with non-equivalent delays** (Table S11)

**
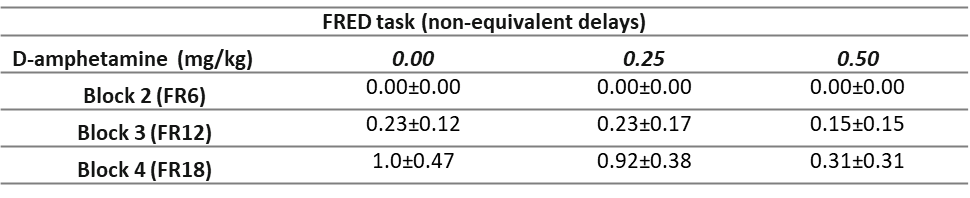
**

**Table S11. Mean number of within-trial omissions recorded in FRED with non-equivalent delays under d-amphetamine treatment as a function of dose and HE/HR response requirement.** A decreasing trend in within-trial omissions was observed after d-amphetamine compared with vehicle treatment in Blocks 3 and 4.

***Effect of d-amphetamine on* *additional performance measures in FRED with non-equivalent delays*** (Table S12)

**
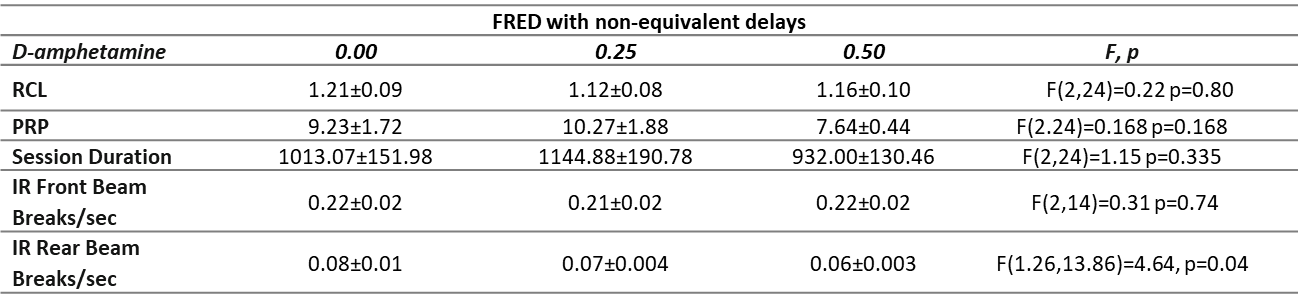
**

**Table S12*.* Summary of additional performance measures in FRED with non-equivalent delays under d-amphetamine treatment.** Significant differences from vehicle were not observed in any of the variables; RCL (Reward Collection Latency), PRP (Post-Reinforcement Pause), Session Duration, and Front and Rear IR (Infrared) Beam Break Rate.

***Impact of pre-feeding on FRED***

Pre-feeding had an effect on the Percentage of HE/HR choices in FRED. A within-subjects repeated measures ANOVA revealed significant effects of Feeding Condition [F(1,12)=99.75, *p<*0.001], Trial Block [F(3,36)=78.30, *p<0.01*] and a Feeding condition x Block interaction [F(3,33)=7.19, *p<*0.00] on this variable. Sidak *post hoc* analysis showed a significant decrease in the Percentage of HE/HR choices in Trial Blocks 3 and 4 (*p=*0.001 and *p<*0.001, respectively) compared to Block 1 when animals were food restricted (baseline). Animals after pre-feeding showed the same pattern of results but a significant decrease was observed in Blocks 2 (*p=*0.03) and 3 (*p=*0.005). The percentage of HE/HR choices was significantly lower in pre-fed animals compared with baseline in Blocks 1-3 (*p=*0.001, *p<*0.001, *p=*0.001, respectively) (Fig. S6A).

The percentage of LE/LR choices in FRED was also affected by feeding condition. A within-subjects repeated measures ANOVA did not reveal a significant effect of Feeding Condition [F(1,12)=0.48, *p=*0.50]. However, a significant effect of Trial Block [F(3,36)=16.50, *p<*0.001] and a Block x Feeding Condition interaction [F(3,36)=50.04, *p<*0.001] on this variable was found. *Post hoc* analysis showed a significant increase in the percentage of LE/LR choices in Blocks 3 and 4 (FR12 and FR18, *p<*0.001) under baseline conditions, suggesting a shift from the HE/HR option to the LE/LR option. However, this pattern of behaviour was not observed when animals were pre-fed. *Post hoc* analysis showed a significant increase in LE/LR choices in Block 2 (FR6, *p<*0.001), but a significant decrease in Blocks 3 and 4 (FR12 and FR18, *p<*0.001). The percentage of LE/LR choices was significantly different between both conditions in all blocks of trials (*p<*0.001) (Fig. S6B).

An overall effect of Trial Block [F(3,36)=63.36 *p<*0.001] and Feeding Condition [F(1,12)=245.22, *p<*0.001], and a significant Block x Feeding Condition interaction [F(1.58,18.91)=11.24 *p=*0.001], was observed on the percentage of omissions. Under baseline conditions, omissions did not significantly change across Trial Blocks. However, when animals were pre-fed, the percentage of omissions significantly increased in Blocks 2 (*p=*0.01), 3 and 4 (*p<*0.001 and *p<*0.001, respectively). Pre-fed animals omitted significantly more than baseline (mild food restriction) in all Trial Blocks (FR6, *p=*0.026; FR12-18, *p*<0.001) (Fig. S6C).


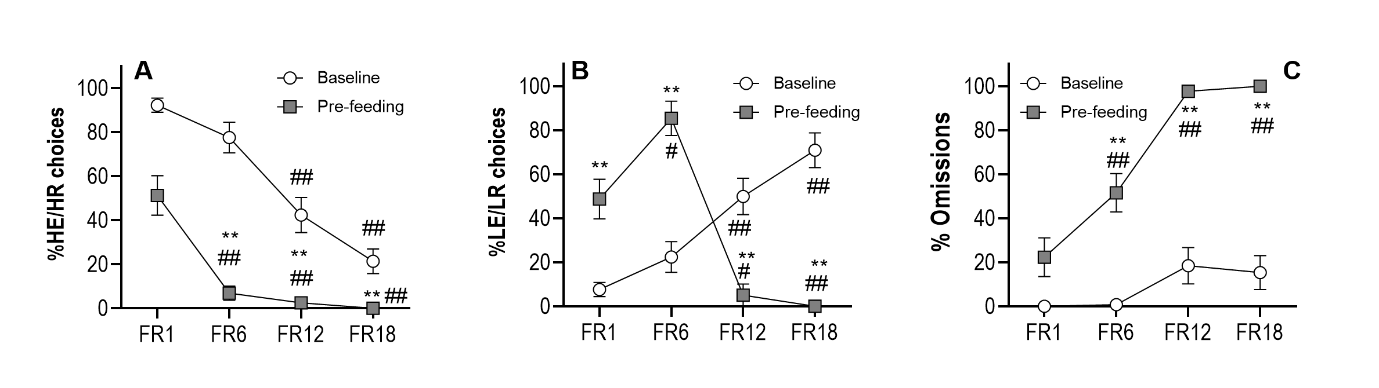


**Fig. S6. Effects of pre-feeding on FRED with non-equivalent delays performance across trial types (defined by HE/HR response requirement)**. Pre-feeding decreased the Percentage of High Effort/High Reward (HE/HR) choices **(A)**, but did not increase the Percentage of Low Effort/Low Reward (LE/LR) choices **(B)**. Pre-feeding increased the Percentage of Omissions **(C)**. **p<0.01 significant difference from baseline. ##p<0.01, #p<0.05 significant effect from Block 1 (FR 1).

***RED but not FRED showed an effort discounting pattern when order of demand was reversed or randomised*** The percentage of HE/HR choices was influenced by effort demand in the RED task when the effort order was changed from ascending to descending (Fig. 5 A, Main Manuscript); this effect was seen as early as Session 1 [F(4,44)=44.08, p<0.001], also in Session 2 [F(4,44)=29.88, p<0.001] and Session 3 [F(4,44)=21.13, p<0.001], consistent with the prediction that in RED mice would not need to remember, infer, or calculate the effort required, and instead could evaluate the effort required immediately on sight of the stimulus. This was also observed when the order of effort (Response Height was randomised (Fig. 5 B, Main Manuscript). Animals decreased the number of HE/HR choices in a Response Height dependent manner in Session 1 [F(2.09,27.23)=32.46, *p<*0.001], Session 2 [F(2.35,30.54)=44.39, *p<*0.001] and Session 3 [F(2.86,37.18)=37.79, *p<*0.001]. The opposite effect was observed on the percentage of LE/LR choices (Fig S7A-B).

However, as shown in FRED with a descending response requirement profile, animals’ preferences were not dependent on effort demand in this task. Block of trials did not have an effect on the percentage of HE/HR choices in any of the three sessions [F(1.85,22.15)=0.49, *p=*0.69; F(2.56,30.66)=0.72, *p=*0.53; F(2.35,28.14)=0.41, *p=*0.70, respectively] nor on the percentage of LE/LR choices (Fig S7C). Statistically significant effects of effort demand on choices are shown in Fig. 5C (Main Manuscript). These findings show that RED, unlike FRED, was robust to the order of presentation of effort conditions even when this order was completely randomised.

**Fig S7.** **The Rearing Effort Discounting (RED) tasks with descending and random effort demand (A-B).** The decrease in percentage of Low Effort/Low Reward (LE/LR) choices as the Response Height decreased. Animals were also sensitive to effort demand when the different Response Heights were displayed in a random order as shown by the increase in the percentage of LE/LR choices when Respond Height was high (**B**). **Fixed Ratio-based Effort Discounting task with descending effort demand C.** This design was not sensitive to changes in the order of effort demand and animals did not show an effort discounting profile (**C**). #p<0.01, ##p<0.05 significant differences from Response Height 0cm.
